# Human tRNAs with inosine 34 are essential to efficiently translate eukarya-specific low-complexity proteins

**DOI:** 10.1093/nar/gkab461

**Published:** 2021-06-14

**Authors:** Adrian Gabriel Torres, Marta Rodríguez-Escribà, Marina Marcet-Houben, Helaine Graziele Santos Vieira, Noelia Camacho, Helena Catena, Marina Murillo Recio, Àlbert Rafels-Ybern, Oscar Reina, Francisco Miguel Torres, Ana Pardo-Saganta, Toni Gabaldón, Eva Maria Novoa, Lluís Ribas de Pouplana

**Affiliations:** Institute for Research in Biomedicine, The Barcelona Institute of Science and Technology, Barcelona, Catalonia 08028, Spain; Institute for Research in Biomedicine, The Barcelona Institute of Science and Technology, Barcelona, Catalonia 08028, Spain; Institute for Research in Biomedicine, The Barcelona Institute of Science and Technology, Barcelona, Catalonia 08028, Spain; Barcelona Supercomputing Centre (BSC-CNS), Barcelona, Catalonia 08034, Spain; Centre for Genomic Regulation, The Barcelona Institute of Science and Technology, Barcelona, Catalonia 08003, Spain; Institute for Research in Biomedicine, The Barcelona Institute of Science and Technology, Barcelona, Catalonia 08028, Spain; Institute for Research in Biomedicine, The Barcelona Institute of Science and Technology, Barcelona, Catalonia 08028, Spain; Institute for Research in Biomedicine, The Barcelona Institute of Science and Technology, Barcelona, Catalonia 08028, Spain; Institute for Research in Biomedicine, The Barcelona Institute of Science and Technology, Barcelona, Catalonia 08028, Spain; Institute for Research in Biomedicine, The Barcelona Institute of Science and Technology, Barcelona, Catalonia 08028, Spain; Institute for Research in Biomedicine, The Barcelona Institute of Science and Technology, Barcelona, Catalonia 08028, Spain; Centre for Applied Medical Research (CIMA Universidad de Navarra), Pamplona 31008, Spain; Institute for Research in Biomedicine, The Barcelona Institute of Science and Technology, Barcelona, Catalonia 08028, Spain; Barcelona Supercomputing Centre (BSC-CNS), Barcelona, Catalonia 08034, Spain; Catalan Institution for Research and Advanced Studies, Barcelona, Catalonia 08010, Spain; Centre for Genomic Regulation, The Barcelona Institute of Science and Technology, Barcelona, Catalonia 08003, Spain; University Pompeu Fabra, Barcelona, Catalonia 08003, Spain; Institute for Research in Biomedicine, The Barcelona Institute of Science and Technology, Barcelona, Catalonia 08028, Spain; Catalan Institution for Research and Advanced Studies, Barcelona, Catalonia 08010, Spain

## Abstract

The modification of adenosine to inosine at the wobble position (I34) of tRNA anticodons is an abundant and essential feature of eukaryotic tRNAs. The expansion of inosine-containing tRNAs in eukaryotes followed the transformation of the homodimeric bacterial enzyme TadA, which generates I34 in tRNA^Arg^ and tRNA^Leu^, into the heterodimeric eukaryotic enzyme ADAT, which modifies up to eight different tRNAs. The emergence of ADAT and its larger set of substrates, strongly influenced the tRNA composition and codon usage of eukaryotic genomes. However, the selective advantages that drove the expansion of I34-tRNAs remain unknown. Here we investigate the functional relevance of I34-tRNAs in human cells and show that a full complement of these tRNAs is necessary for the translation of low-complexity protein domains enriched in amino acids cognate for I34-tRNAs. The coding sequences for these domains require codons translated by I34-tRNAs, in detriment of synonymous codons that use other tRNAs. I34-tRNA-dependent low-complexity proteins are enriched in functional categories related to cell adhesion, and depletion in I34-tRNAs leads to cellular phenotypes consistent with these roles. We show that the distribution of these low-complexity proteins mirrors the distribution of I34-tRNAs in the phylogenetic tree.

## INTRODUCTION

Transfer RNAs (tRNAs) are essential components of the translation machinery that physically connect amino acids to their cognate nucleotide triplets (anticodons) according to the Genetic Code. Regulation of tRNA pools is a well-known adaptive mechanism that acts in combination with codon usage to implement translational responses to internal or external cues (1). Codon-anticodon interactions are optimized and modulated by post-transcriptional chemical RNA modifications such as inosine (2,3) that are species-specific, and vary greatly across the phylogenetic tree (4,5). Thus, although the genetic code is essentially universal, the mechanisms that decode it are not.

**Figure 1.**
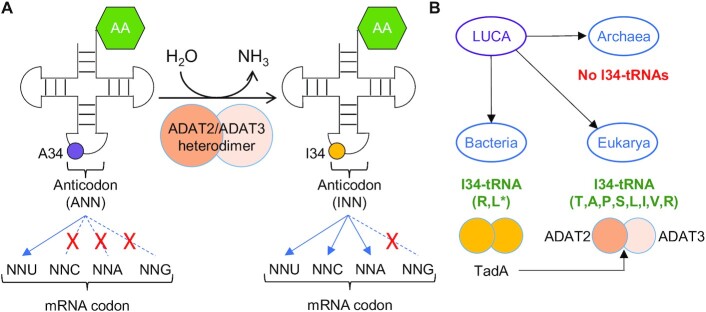
(**A**) Schematic representation of adenosine deamination to inosine at position 34 of eukaryotic tRNAs (A34-to-I34 editing), and the preferential codon:anticodon pairing for unmodified (left) and modified (right) tRNAs. ‘AA’: amino acid Thr, Ala, Pro, Ser, Leu, Ile, Val or Arg (TAPSLIVR). The anticodon region within the cloverleaf structure of tRNAs is indicated (see also Supplementary Figure S2B for a detailed example of anticodon structure). (**B**) I34 in Bacteria is catalysed by the homodimeric TadA enzyme, from which the eukaryotic ADAT2/ADAT3 heterodimeric enzyme evolved. I34-tRNAs are absent in Archaea, present in two substrates in Bacteria (*to date bacterial I34-tRNA^Leu^ has been reported only on *Oenococcus oeni* (10)), and present in eight substrates in Eukarya.

Inosine at position 34 of the tRNA (I34; first nucleotide of the tRNA anticodon) is produced in Bacteria and Eukarya through the deamination of adenosine (A34) (6,7) (Figure 1). Whereas tRNAs with A34 can only efficiently decode U-ended codons, tRNAs with I34 can decode U-, A- and C-ended codons (8) by wobble pairing (Figure 1A). In Bacteria I34 is produced by the homodimeric enzyme tRNA adenosine deaminase A (TadA), and, in Eukarya, by the heterodimeric adenosine deaminase acting on tRNA (ADAT). ADAT evolved from TadA early in eukaryotic evolution, through a duplication of the bacterial *tadA* gene that gave rise to the two genes coding for the two ADAT subunits (*ADAT2* and *ADAT3*) (6,9) (Figure 1B). In Bacteria, I34 can be found in two different tRNAs (almost universally on tRNA^Arg^_ACG_ and rarely on tRNA^Leu^_AAG_) (10,11). In contrast, eukaryotic I34 is found in eight tRNAs (tRNA^Thr^_AGT_, tRNA^Ala^_AGC_, tRNA^Pro^_AGG_, tRNA^Ser^_AGA_, tRNA^Leu^_AAG_, tRNA^Ile^_AAT_, tRNA^Val^_AAC_ and tRNA^Arg^_ACG_) (6,10,12–20) that are highly enriched in eukaryotic genomes (10,21) (Figure 1B). Interestingly, additional A34-tRNAs have been reported in Bacteria but they are not modified to I34-tRNAs, suggesting that the expansion of I34-tRNAs began with the emergence of unmodified A34-tRNAs (10).

In Bacteria, G34-tRNAs (with the exception of tRNA^Arg^, see above) are globally used as the major isoacceptors to decode threonine, alanine, proline, serine, leucine, isoleucine, valine and arginine (TAPSLIVR). In Eukarya, however, the expansion of eukaryotic I34-containing tRNAs (I34-tRNAs) replaced G34-tRNAs as the preferred mechanism to decode these amino acids (4,5,10,22–25), and played a major role in defining the structure and codon composition of eukaryotic genomes (21). Structure-based hypotheses have been put forward to explain the eukaryotic expansion of I34-tRNAs that affected all three-, four-, and six-codon boxes of the Genetic Code (with the exception of glycine) (26,27). However, the selection forces that drove this phenomenon at the root of eukaryotic evolution remain unclear. Nevertheless, it stands to reason that selection of I34-tRNAs was linked to the mechanisms involved in the translation of codons for TAPSLIVR.

Protein regions of low amino acid diversity are commonly referred to as ‘low-complexity’ domains (28). Low-complexity domains may or may not be structured, depending on their amino acid composition (29–32), and are often important components of structural, extracellular matrix (ECM) and cell adhesion proteins (33–37). Translating low-complexity coding sequences is a challenge because their highly biased codon composition can slow down translation (38), induce frameshifts leading to mistranslation (39), or cause ribosome stalling and translational arrest (40). Both universal and species-specific adaptations exist to overcome these challenges and expand the protein repertoire (33,40–42), and it is possible that the selection and enrichment of I34-tRNAs in eukaryotic genomes is connected to the contribution of these tRNAs to the efficient translation of low-complexity TAPSLIVR-rich proteins.

Here, we investigate the functional relevance of I34-tRNAs in human cells. The complete elimination of I34 in tRNAs is lethal in all the species where this has been attempted (6,14,16,17,19,20). Thus, generating a cellular model completely devoid of I34-tRNAs is not possible. However, we find that partial I34-tRNA depletion is tolerated in human cells and does not affect translational efficiency or accuracy at global scale. Under these experimental conditions, pathways particularly sensitive to I34-tRNA levels may be identified. Indeed, we find that the impact upon gene translation of a partial reduction in I34-tRNA levels is codon-dependent, and mostly affects low-complexity TAPSLIVR-rich proteins that are prevalent in functional categories linked to cell-cell interactions and ECM-associated pathways. Chief among these proteins are polypeptides containing mucin-like domains. Consistently, I34-tRNA depletion results in abnormal cell morphology and impaired adhesion caused by the deficient translation of membrane proteins exposed to the extracellular environment.

Phylogenetic analyses reveal that TAPSLIVR-rich low-complexity proteins are essentially absent in Bacteria and Archaea, but are abundant in eukaryotes. Moreover, and consistent with their roles in cell adhesion, we find that these proteins are significantly enriched in multicellular species. Our results indicate that I34-tRNAs improve the translation efficiency of genes with highly biased codon compositions that would, otherwise, be inaccessible to the translation apparatus. We propose that the eukaryotic expansion of I34-tRNAs, and of related codons in eukaryotic genomes, was driven by the increase in proteome diversity afforded by the modified tRNAs.

## MATERIALS AND METHODS

### Cell lines and cell culture

Human cell lines HEK293T (female; RRID:CVCL_0063), HeLa (female; RRID:CVCL_0030) and HT-29 M6 (female; RRID:CVCL_G077) were maintained in Dulbecco's modified Eagle's medium (DMEM) (41966029, Thermo Fisher), and NCI-H292 (female; RRID:CVCL_0030) cells were maintained in Roswell Park Memorial Institute (RPMI) 1640 Medium (ATCC modification) (A1049101, Thermo Fisher). All media were supplemented with 10% fetal bovine serum (FBS) (10270106, Thermo Fisher), 100 U/ml Penicillin–Streptomycin (15140122, Thermo Fisher) and 25 μg/ml plasmocin (ant-mpp, InvivoGen); herein ‘Full media’. Cells were grown at 37°C in a humidified atmosphere with 5% CO_2_ (37°C/5% CO_2_), and were periodically checked for mycoplasma contamination by PCR. The cell line HT-29 M6 was a gift from Dr Eduard Batlle (IRB Barcelona), and the cell line NCI-H292 was provided by Dr Ana Pardo (CIMA, University of Navarra).

### Generation of CRISPR-ADAT KD cell lines

Guide strands were designed using public resources (http://crispr.mit.edu) (see Supplementary Table S1 for detailed oligonucleotide sequences), and were cloned into px330 SV40-GFP vector (gift from Dr Eduard Batlle, IRB Barcelona) (43) as described in (44).

HEK293T cells growing in six-well plate format were transfected with 3 μg px330 SV40-GFP (CTRL), px330 SV40-GFP ADAT2 (ADAT2 KD) or px330 SV40-GFP ADAT3 (ADAT3 KD) constructs using lipofectamine 2000 (L2K) (11668027, Thermo Fisher) following the manufacturer's protocol (250 μl plasmid/lipid reaction in 2 ml DMEM Full Media). Forty-eight hours later, GFP-positive cells were sorted using a FacsAria I SORP sorter (Beckton Sickinson). Sorting on 96-well plates was done using an ACDU system, and one cell per well was sorted in wells containing 100 μl DMEM Full Media (see Supplementary methods). Out of 96 clones analysed per cell line, 55 and 74 were inviable when they were derived from px330 SV40-GFP ADAT2 or px330 SV40-GFP ADAT3 treated cells, respectively. Out of the viable clones, none presented a full KO of the targeted gene, suggesting that full ablation of ADAT2 or ADAT3 is lethal in this cell line. 100% of the single cell seeded clones derived from px330 SV40-GFP treated cells (CTRL) were viable. DNA edition was confirmed by sequencing.

### Cell line generation by lentiviral infection

shCV and shADAT2 stable cell lines were generated as previously described (17). Plasmid for hADAT2 over expression was generated by Gateway cloning system following the manufacturer's protocol (hADAT2-pDONR221) using specific oligonucleotides (Supplementary Table S1; and see Supplementary methods). hADAT2 gene was amplified from HEK293T cDNA. hADAT2-pLenti construct was generated by performing an LR reaction using hADAT2-pDONR221 and pLenti vector (Adgene plasmid 19068: pLenti PGK Puro DEST W529-2) following the manufacturer's protocol. Plasmids for DOX-inducible shRNA expression (shNonTarget and shADAT2) were generated by cloning the respective sh sequences (see Supplementary methods) into pTRIPZ vector (Thermo Scientific Open Biosystems Expression Arrest TRIPZ Lentiviral shRNAmir) following the design guidelines reported previously (45).

All shCV, shADAT2, pLenti-hADAT2, and DOX-inducible shRNA cell lines were generated by lentiviral infection using the aforementioned plasmids as described in (17) (see also Supplementary methods). For the transduction of NCI-H292 cells, viral supernatants obtained from HEK293T cells were collected, cleared with a 0.45 μm filter and concentrated by ultracentrifugation through a 20% sucrose cushion at 26 000 g for 2 h at 4°C using a Beckman SW-28 rotor. Purified lentiviral particles were re-suspended in PBS, aliquoted and stored at −80°C. Lentiviral titer was determined using QuickTiter Lentivirus Quantitation Kit (Cell Biolabs, VPK-107). NCI-H292 cells were infected at a MOI of 6 for 24 h, and puromycin at 2 μg/ml was added to culture medium for selection of transduced cells two days later.

### Protein extraction

Unless stated otherwise, all protein extractions were performed with ‘RIPA buffer’: 50 mM Tris pH 7.5, 150 mM NaCl, 1% NP-40, 0.1% SDS, 1× ‘cOmplete’ EDTA-free Protease Inhibitor Cocktail (PIC) (11873580001, Merck) (see Supplementary methods). Quantification of protein extracts was performed using Pierce BCA Protein Assay Kit (23227, Thermo Fisher) and measuring absorbance at 562 nm with a Synergy HTX Multi-Mode reader (BioTek). For differential extraction of RIPA-soluble and RIPA-insoluble proteins, a pellet of 16 × 10^6^ cells was re-suspended in 250 μl of RIPA buffer, and RIPA-soluble fractions were obtained. The remaining pellet was washed once with 1 ml RIPA buffer and was then re-suspended in 250 μl of ‘Solubilisation buffer’: 50 mM Tris pH 7.4, 150 mM NaCl, 50 mM DTT, 2% SDS, 8 M urea, 1× PIC. The re-suspended pellet was incubated for 10 min at 95°C, centrifuged at maximum speed for 2 min at room temperature (RT°), and supernatant was recovered (RIPA-insoluble fraction). For Figure 3E, 250 μl of ‘insoluble protein loading buffer 2×’ (100 mM Tris pH 6.8, 0.1% Bromophenol blue, 20% glycerol) was added to each RIPA-soluble and RIPA insoluble fractions. Then, 20 μl of each sample was resolved by 10% PAGE and the gel was stained with BlueSafe (MB15201, NZYtech).

### RNA extraction

Total RNA was isolated from cells with TRIzol (15596026, Thermo Fisher) and ethanol re-precipitated as described (17). For RNA extraction from high polysome fractions, samples were combined and concentrated using an Amicon Ultra-15 mL 100K Da (UFC910024, Merck) to a volume of approximately 200 μl and RNA was extracted using 500 μl TRIzol LS (10296010, Thermo Fisher) following the manufacturer's protocol. Extracted RNA was quantified using a Nanodrop ND1000 spectrophotometer (Thermo Fisher). RNA integrity was evaluated with a 2100 Bioanalyzer Instrument (Agilent).

### Western blots

Western blotting was performed by standard procedures as previously described (46). See Supplementary methods for details on antibodies used in this study. Blots were developed using an Odyssey Fc Imaging System (LI-COR) and analysed using Image Studio Lite v5.2. Raw Odyssey FC image files available upon request.

### Real-Time quantitative PCR

RT-qPCR was performed as previously described (17,46) in a StepOnePlus Real-time PCR System (Applied Biosystems). Details on primers used are shown in Supplementary Table S1 (17,47–49).

### Analyses of tRNA-Seq datasets

Inosine and 1-methylinosine quantifications by tRNA-Seq were performed as previously described (17), except that reads were aligned against the human reference genome hg38. Quantification of tRNA gene expression at tRNA isodecoder level was performed with DESeq2 v1.18 (50) as previously described (51). Datasets used in this study GSE114904 (51) and PRJEB8019 (17).

### Pulse-chase analyses

Pulse-chase experiments were performed with cells at approximately 80% confluence. Growing media was removed, cells were washed twice with PBS, and incubated at 37°C/5% CO_2_ for 30 min in Starvation media: DMEM No Cys, No Met, No Glu (21013024, Thermo Fisher), 10% FBS, 4 mM l-glutamine (25030024, Thermo Fisher). Media was then removed and cells were incubated for 30 min at 37°C/5% CO_2_ with Pulse media: Starvation media containing 300 μCi/ml of ^35^S-Met/^35^S-Cys (EasyTag™ EXPRESS35S Protein Labeling Mix, NEG772007MC, Perkin Elmer) or ^35^S-Met (NEG 009L005 MC, Perkin Elmer) and 0.2 mM L-Cys (non-radioactive) (C6852, Merck). Cells were washed twice with PBS and were incubated for 5 min at RT° with Chase media: DMEM Full media, 5 mM L-Cys (non-radioactive), 5 mM L-Met (non-radioactive) (M9625, Merck). Cells were then washed twice with PBS and harvested with PBS. When cycloheximide (CHX) treatments were required, Starvation, Pulse and Chase media contained 100 μg/ml CHX (C4859, Merck). Proteins were extracted with RIPA buffer and 10 μg of obtained proteins were resolved by 10% SDS-PAGE. The gel was stained with Coomassie (A1092, Panreac AppliChem), dried using a Slab Gel Dryer GD2000, and exposed to a Typhoon Screen for radioactivity detection.

Quantitative metabolic labeling was performed as previously described (52). Pulse-labeling medium contained 50 μCi/ml of ^35^S-Met/^35^S-Cys (EasyTag™ EXPRESS35S Protein Labeling Mix, NEG772007MC, Perkin Elmer). Cells were incubated with pulse-labeling medium for 15, 30 and 60 min, were washed and collected as described (52). Cell pellets were resuspended in 100 μl ice-cold PBS and 15 μl of cell suspension were spotted on 2.5 cm glass microfiber filter disks (Whatman GF/C; WHA1822025) (to measure total radioactivity) or to perform TCA precipitation (to measure TCA-precipitable label) as described (52). When CHX treatments were required, Starvation media, Pulse-labeling media and PBS contained 100 μg/ml CHX (C4859, Merck). Scintillation counting was measured in a Tri-Carb 2900 TR (Perkin Elmer) as described (52).

### Analyses of cell growth

1 × 10^6^ cells in 8 ml Full Media (time point Day 0) were seeded on a 10 cm Petri dish. Two days later, cells were washed once with PBS, and harvested with 2 ml Trypsin-EDTA (0.05%) (25300054, Thermo Fisher) that was later quenched with 2 ml Full Media. Total number of cells was counted (time point Day 2) using a Countess Automatic Cell Counter (Invitrogen). Then, 1 × 10^6^ harvested cells were plated on a new 10 cm Petri dish and the process was repeated up until the last time point. Results represent the cumulative counting of cells from Day 0 to the last time point.

### Cell cycle analyses

Cells were synchronised by a double thymidine block: 2 mM thymidine (T1895, Merck) in DMEM Full Media for 13 h, release (media without thymidine) for 8 h, and second block for 17 h. Determination of cell cycle stages were performed in an Epics Cyan ADP flow cytometer (Beckman Coulter) as previously described (46).

### Cellular treatments with stress reagents

1 × 10^6^ cells in DMEM Full Media were seeded in six-well plate format. Forty-eight hours later, media was replaced by 2 ml DMEM Full Media containing a either 700 μg/ml hygromycin B (HygroB) (10687010, Thermo Fisher), 700 nM emetine (E2375, Merck), 100 μg/ml blasticidin S (BlaS) (R21001, Thermo Fisher), 100 μg/ml cycloheximide (CHX), 50 mM CaCl_2_ (1.02391, Merck), 0.45 M Sucrose (84097, Merck), or DMEM only (no FBS; starvation). Cells were visualised in an Eclipse Ts2-FL microscope (Nikon). Results depicted on Figure 5A were obtained at 2 h (Sucrose), 18 h (BlaS and CHX), 20 h (Emetine and HygroB) and 24 h (CaCl_2_ and starvation) of treatment. Every condition had its own ‘untreated control’ per cell line; Figure 5A shows a representative untreated control. Activation of the UPR (Figure 3D) was performed by treating cells with 2 μM thapsigargin (T9033, Merck) for 3 h. Proteins were then extracted with RIPA buffer containing 1 mM Na_3_VO_4_, 5 mM Na_4_P_2_O_7_ and 50 mM NaF to retain their phosphorylation status.

### Cell viability assays

To prevent ADAT KD cells to detach upon treatments with stress reagents, 96-well culture plates were coated with 100 μg/ml rat-tail collagen type I (A1048301, Thermo Fisher). 1 × 10^4^ cells were seeded per well and 48 h later were treated with stress reagents for 12 h. Cell viability was measured with reagent WST-1 (5015944001, Merck) following the manufacturer's protocol in a Synergy HTX Multi-Mode reader (BioTek).

### Cellular adhesion to ECM components

Cells were harvested using Trypsin and re-plated in 175 cm^2^ flasks. Twenty-four hours after plating, cells were washed once with pre-warmed PBS and harvested with pre-warmed PBS/2 mM EDTA (131026.1209, Panreac Quimica). 1.5 × 10^5^ harvested cells in 100 μl Assay Buffer were plated for 2 h at 37°C/5% CO_2_ on an ECM Array Plate (ECM cell adhesion array kit colorimetric, ECM540, Merck). Wells in ECM Array Plates are pre-coated with individual ECM components to test the binding preferences of seeded cells. The following steps of the assay were performed as described by the manufacturer's protocol. For control experiments depicted in Supplementary Figure S3E, cells were harvested with Trypsin instead of PBS/2 mM EDTA.

### Polysome profiling

Cells growing in 175 cm^2^ flasks were trypsinised and plated in three 10 cm Petri dishes. Each dish contained 1 × 10^7^ cells (for experiments carried out 24 h after plating; Figure 6A) or 1 × 10^6^ cells (for experiments carried out 72 h after plating; Supplementary Figure S4A). At 24 or 72 h after plating, cells were lysed following a protocol adapted from (53). All solutions were prepared fresh on the day to be used. Cells in each Petri dish were treated with 7 ml of 100 μg/ml CHX in DMEM Full Media at 37°C/5% CO_2_ for 3 min. Cells were then washed with 4 ml ice-cold PBS containing 100 μg/ml CHX (PBS/CHX). PBS/CHX was removed, cells from all three Petri dishes were harvested by scrapping, combined to generate a single cell lysate, and kept on ice at all times. Cells were centrifuged at 1000 × g for 5 min at 4°C and the supernatant was discarded. Cell pellets were gently washed (one pipette ‘up and down’ stroke) with 1 mL PBS/CHX, and were centrifuged and the supernatant was removed as before. Cell pellets were re-suspended (five pipette strokes) in 500 μl ‘Polysome extraction buffer’ (PEB) (20 mM Tris pH 7.4, 100 mM KCl, 10 mM MgCl_2_, 0.5% NP-40, 2 mM DTT, 100 μg/ml CHX, 100 U/ml RNasin (N2615, Promega), 1× PIC). Cells were incubated on ice for 10 min, vortexing briefly every 2 min. Cell lysate was then centrifuged at maximum speed for 10 min at 4°C and supernatant (approximately 600 μl) was recovered. 10% of this lysate was used for total RNA extraction, and the rest was used to obtain polysome profiles. Polysome profiling was carried out as described in (54) using a 10–50% linear sucrose gradient, with minor modifications (see Supplementary methods). P/M ratios were obtained from three independent replicates, after integrating the area under the curve of monosomes (peak corresponding to the 80S fraction) and polysomes (peaks corresponding to low- and high-polysome fractions).

### RNA-Seq

Library preparations for RNA-Seq studies (total RNA and HP fractions, in biological triplicates, for both HEK293T CTRL and HEK293T ADAT2 KD cell lines) were prepared using the TruSeq mRNA library preparation kit (single indexes set A; 20020492, Illumina), following manufacturer's recommendations. Libraries were indexed, pooled, and then sequenced in a NextSeq Flow Cell machine as 2 × 150 bp paired-end reads. Datasets have been deposited at NCBI GEO, accession GSE150860.

Reads were aligned to the human genome (hg38) using STAR 2.3.0e with default options. Reads counts at gene level were generated with the featureCounts function from the Rsubread package version 1.28.1 using options annot.inbuilt = ‘hg38’, isPairedEnd = TRUE, requireBothEndsMapped = TRUE, checkFragLength = TRUE. Only protein coding genes (Ensembl biomart v97 July 2019) having >10 reads in at least half of the samples were considered for differential expression analyses. DESeq2 1.18 was used to detect differentially expressed genes with default options and using the following thresholds: Benjamini–Hochberg adjusted *P*-value < 0.1, |FC| > 1.5. The ROAST method (55) was used to perform Gene Set Enrichment Analyses using the MaxMean statistic (56). All gene set mapping was performed at Gene Symbol level (org.Hs.eg.db v3.0.0). Statistically significant categories were defined as those having an adjusted *P*-value < 0.05.

Statistical significance of the differences between empirical distributions of global TE was computed using the mded package version 0.1–2. (57) (Figure 6C). Downregulated genes for the interaction analysis (HP ADAT2 KD/HP CTRL)/(Total RNA ADAT2 KD/Total RNA CTRL) were detected using DESeq2 with FC HP versus Total < 1.5; *P*-value <0.05. Statistical significance of the enrichment of transcripts encoding low-complexity TAPSLIVR-rich proteins among those down-regulated in the interaction analysis was assessed via Fisher Exact Test. (Supplementary Table S4). Enrichment in proportion of transcripts encoding low-complexity TAPSLIVR-rich proteins with decreased TE in ADAT2 KD cells, among transcripts with TE CTRL >1.5 was assessed via permutation test (*n* = 31, *B* = 10000) (Figure 8B). *P*-values are computed as the proportion of permutations with more extreme statistics than the observed.

### Construction of ADAT eGFP reporters

eGFP ADAT and eGFP nonADAT sequences flanked by EcoRI, XhoI and XbaI restriction sites (5-end) and PmeI, AgeI and EcoRI restriction sites (3′-end) were ordered from GenScript (GenScript HK Inc) and were cloned into a custom pLV-CMV-SV40-Puro plasmid. Correct sequence insertion for all constructs was verified by Sanger sequencing using the CMV-F universal primer (GATC Biotech). Details on eGFP ADAT sequence and eGFP nonADAT sequence are depicted in Supplementary methods. The eGFP open reading frame contains 239 codons, 88 of which encode for TAPSLIVR and are uniformly distributed across the gene. Thus codon differences between the reporters affected 36.8% of the eGFP sequence. Importantly, these differences did not significantly affect the Codon Adaptation Index (CAI) of the genes (CAI-eGFP ADAT = 0.761; CAI-eGFP nonADAT = 0.759). In addition, since all TAPSLIVR codons were modified to either C-ended (eGFP ADAT) or G-ended (eGFP non-ADAT) triplets, the overall GC content of the genes remained unaltered (GC content for both eGFP sequences = 61.8%). Conservation of CAI and GC-content is important to rule out potential non-I34-tRNA dependent effects on eGFP expression.

### Evaluation of ADAT eGFP production

Cells growing on 6-well plate format at approximately 80% confluence were transfected with 2.5 μg of eGFP ADAT or eGFP nonADAT plasmids using L2K (250 μl plasmid/lipid reaction in 2 ml DMEM Full Media), following the manufacturer's protocol. Negative control cells (‘L2K only’) received the same amount of lipid formulation without plasmids. Proteins were extracted with RIPA buffer 48 h after transfection and western blots were carried out as described above. For FACS analyses, 24 h after lipofection, cells were washed twice with PBS and the cell suspension was analysed in a Cytomics FC500 MPL flow cytometer (Beckman Coulter) (see also Supplementary methods). Data was analysed with Summit v4.3 or FlowJo v10.5.3.

### *In silico* detection of low-complexity TAPSLIVR-rich genes

In this work we refer to ‘low-complexity regions’ as sections of a protein sequence bearing low amino acid diversity, a widely used definition (28). Based on the concept of a TAPSLIVR-rich region defined by Rafels-Ybern *et al.* (23), we consider that a low-complexity region is rich in TAPSLIVR if at least 80% of its amino acids (in any combination) belong to the TAPSLIVR category. Bioinformatics identification of low-complexity TAPSLIVR-rich regions were performed on the Human CCDS release 22 (14 June 2018), using a running window strategy as previously described (23), but the window size was modified to include regions of 30 or more amino acids to evaluate a larger number of proteins. Based on the reported *H. sapiens* codon usage (58), we applied a threshold of 65.743% to define a genetic sequence as significantly enriched in ADAT-dependent codons. Gene ontology analyses were performed with DAVID (Database for Annotation, Visualization and Integrated Discovery) v6.8 (59) using default options. Statistically significant categories were defined as those with a FDR <0.25 and Benjamini-Hochberg adjusted *P*-value <0.05. For the Functional Annotation Clustering sets, only those with an Enrichment Score >4 were included.

### Construction of ADAT luciferase reporters

SDC3-RLuc and SDC3(G-end)-RLuc plasmids were generated using the backbone vector psiCHECK-2 (C8021, Promega). The psiCHECK-2 RLuc gene was PCR amplified from the vector and the desired portion of SDC3 was PCR amplified from HEK293T cDNA (or from a custom made SDC3(G-end) sequence, GeneArt, Life Technologies). A linker (reported in Promega's NanoLuc reporter plasmids) that serves as a spacer between the SDC3 section and the RLuc gene was present in the reverse (RVR) primer used to amplify SDC3/SDC3(G-end). The obtained SDC3/SDC3(G-end) and RLuc products were ligated and inserted into the psiCHECK-2 vector resulting in replacement of the original RLuc gene. Oligonucleotides used are depicted in Supplementary Table S1 (see Supplementary methods for further details).

### Luciferase assays

6 × 10^4^ cells in 100 μl DMEM Full Media per well were plated in a 96-well black plate with clear bottom (CLS 3603, Merck), and were lipofected with 100 ng plasmids on the next day following the manufacturer's protocol (50 μl of plasmid/lipid reaction in 100 μl DMEM Full Media per well). Cells were left at 37°C/5% CO_2_ until luciferase measurements. Luciferase activity was monitored using the Dual-Glo Luciferase Assay System (E2920, Promega), following the manufacturer's protocol using a MicroLumat Plus LB96V luminometer (Berthold).

### Purification of reporter proteins

Reporter proteins were purified following standard procedures. SDC3-RLuc and SDC3(G-end)-RLuc were purified using magnetic Dynabeads Protein A (10002D, Thermo Fisher) incubated with a Renilla luciferase antibody (PA5-32210, Thermo Fisher) and cross-linked with 5 mM BS^3^ (21580, Thermo Fisher) in Conjugation Buffer (20 mM NaP, 150 mM NaCl). eGFP ADAT and eGFP nonADAT were purified using Protein G sepharose beads (17-0618-01, VWR) incubated with Green Fluorescent Protein antibody (DSHB-GFP-12A6, Developmental Studies Hybridoma Bank) using magnetic Dynabeads Protein A (10002D, Thermo Fisher), following the manufacturer's protocol. Purified proteins were visualized by SDS- PAGE, and confirmed by western blotting. See Supplementary methods for further details.

### Mass Spectrometry analyses

Protein samples were reduced, alkylated and overnight tryptic digested (60). Digested peptide mixtures were desalted and clean-up using polyLC C18 and strong cation-exchange (SCX) filters. Samples were subject to nano-LC–MS/MS analysis. The nanochromatographic system used was either a Nanoacquity (Waters) or a Dionex Ultimate (Thermo Scientific). The Advion Triversa NanoMate (Advion Bioscieneces) was used as the nanosource and it was fitted on an LTQ-FT Ultra (Thermo Fisher) or an Orbitrap Fusion Lumos mass spectrometer (Thermo Fisher). The mass spectrometer was operated in a DDA mode, with survey scans acquired at 120 k and MS2 scans at 30 k in the orbitrap or IT resolution.

Data processing was performed with Proteome Discoverer software v2.1 or Bioworks v3.1.1 SP1 (Thermo Fisher) using Sequest HT search engine and SwissProt HUMAN, contaminants and the proteins of interest (SDC3-RLuc or eGFP) fasta databases. Search parameters included trypsin as enzyme, carbamidomethylation in cysteine as fixed modification and oxidation in methionine as variable modification. Peptide mass tolerance was 10 ppm and the MS/MS tolerance was 0.6 Da (MS2 in the IT) or 0.02 Da (MS2 in the Orbitrap). Peptides with FDR <1% were considered as positive identifications with a high confidence level.

To identify possible mistranslation in SDC3-RLuc we performed *de novo*, database and homology searches using PEAKS v8.5 with search parameters as described above. *De novo* score (ALC %) threshold was set to 15 and peptide hit threshold (-10logP) was 30.0. *De novo* hits that did not match any database or homology searches and that have an ALC >90% were used in a BLAST (The Basic Local Alignment Search Tool) search against SDC3-RLuc protein in order to find regions of local similarity between sequences and highlight possible mutations.

Whole proteomics analyses was performed in biological triplicates. HEK293T CTRL and ADAT2 KD cells growing in 10 cm Petri dishes and at ∼80% confluence were washed once with PBS and harvested by cell scrapping with 0.5 ml proteomics extraction buffer (0.1 M Tris–HCl pH 7.5; 0.1 M DTT; 4% SDS). The lysate was further processed through a 20 G needle 20 times and then through a 15 G needle 15 times to shear DNA, and was quantified using the Bradford reagent (B6916, Merck). 100 μg of protein sample were then processed following the filter-aided sample preparation (FASP) method (61). Before trypsin digestion, urea buffer was removed and exchanged with triethylammonium bicarbonate (TEAB) buffer. Digested solutions were acidified to a final concentration of 0.1% formic acid. Samples were then dried in a speedvac and reconstituted in 46 μl TEAB 500 mM. 30 μl of sample was labeled with iTRAQ Reagent-8PLEX Multiplex Kit (4390812, Sciex) following the manufacturer's protocol. In addition, 11 μl of each sample were combined to generate 2 pools of all samples and were also labeled. The combined iTRAQ-labeled sample was cleaned up using polyLC C18 and SXC filters. Cleaned-up combined iTRAQ-labeled sample was fractionated using the Pierce High pH Reversed-Phased Peptide Fractionation kit (84868, Thermo Scientific) following the manufacturer's protocol. Fractions were dried with a speedvac and reconstituted in 58.8 μl 2% acetonitrile and 0.1% formic acid. LC–MS/MS analysis was done with the Advion Triversa Nanomate (Advion Bioscieneces) fitted on an Orbitrap Fusion Lumos mass spectrometer (Thermo Fisher). Data processing was performed with Proteome Discoverer v2.1 as described above.

iTRAQ reporter ion intensities were used for protein quantifications. Contaminant sequences were removed. Unique and razor peptides with an average reporter ion signal to noise >1 were considered for further quantitative and statistical analysis. Within each iTRAQ experiment, peptide quantitation was normalized by summing the abundance values for each channel over all peptides identified within fractions. For each protein a linear model was fitted with or without random effects depending on available data. Condition was selected as fixed effect and peptide, fraction and replicate were set as random effects. Model fitting was accomplished with the lme4 R package version 1.1–23. Differentially expressed proteins were defined as those with |FC| > 1.5 and Benjamini & Hochberg adj. *P*-value <0.1.

The mass spectrometry proteomics data have been deposited to the ProteomeXchange Consortium via the PRIDE (62) partner repository with the dataset identifier PXD025024.

### Evaluation of MUC5AC production

For induction of mucous cell differentiation, NCI-H292 shCV and shADAT2 cells were seeded in six-well plates at 6 × 10^5^ cells per well and incubated at 37°C/5% CO_2_ in RPMI Full Media containing 2 μg/ml puromycin for 3 days or until confluent. Cells were then washed with PBS and incubated in puromycin-free fresh media containing either 50 nM amphiregulin (AREG) (A7080, Thermo Fisher) or PBS for 2 days. Treatment was then repeated for another 2 days. Immunofluorescence was performed on cells grown on glass coverslips. Cells were fixed with 4% paraformaldehyde for 15 min at RT°, washed twice with PBS, and permeabilised in Blocking buffer (0.3% Triton-X100, 1% BSA, 1X PBS) for 20 min at RT°. Cells were stained with MUC5AC (45M1) primary antibody (MA1-38223, Thermo Fisher) diluted 1:200 in blocking buffer overnight at 4°C, cells were then washed twice with PBS and incubated with 1:400 dilution of Anti-Mouse Alexa Fluor 555-conjugated secondary antibody (A-31570, Thermo Fisher) in the dark for 1 h at room temperature. Slides were then stained with DAPI (D9542, Merck), and were mounted with Vectashield (H-1000, Vector Laboratories). Images were acquired with a Zeiss LSM 780 confocal microscope and analysed using ImageJ software (63). All image adjustments were applied to all images equally for direct comparison. Quantification of MUC5AC signal was performed on at least 31 different images per condition taken with a Plan-Apochromat 10×/0.45 M27 objective, and using custom-made macros in ImageJ.

For FACS analyses, NCI-H292 cells were harvested using Ca^+2^-free PBS/0.02% EDTA to avoid enzymatic degradation of extracellular proteins, washed in PBS, and re-suspended in Flow cytometry buffer (PBS containing 0.1% (w/v) saponin (S7900, Merck), 1% (w/v) sodium azide (S2002, Merck), and 10% FBS). Aliquots of 5 × 10^5^ cells were then stained with MUC5AC (45M1) primary antibody (dilution 1:200 in flow cytometry buffer) at 4°C for 30 min, washed twice in flow cytometry buffer, and incubated with 1:250 dilution of Anti-Mouse Alexa Fluor 488-conjugated secondary antibody (A-21202, Thermo Fisher) in the dark at 4°C for 30 min. Cells were then washed twice in flow cytometry buffer, re-suspended in cold PBS and analysed on a FACSAria Fusion flow cytometer (BD Biosciences). Cells stained only with secondary antibody were used as negative control to set the gate. Representative plots showing the gating strategy are shown in Supplementary Figure S6D and E.

### Homology search

The search for homologous sequences was performed using the OMA database (64) of 152 archaeal, 1674 bacterial, and 462 eukaryotic genomes. For consistency, we used the same version of the *Homo sapiens* genome (see 'In silico detection of low-complexity TAPSLIVR-rich genes'). A BlastP (v2.5.0) (65) search was performed with each human protein containing low-complexity TAPSLIVR-rich regions (2218 proteins: TAPSLIVR-set), and with the rest of the human proteins, against the OMA database. The number of accepted hits was 10 000. Blast results were filtered using an e-value cut-off of 0.01 and an overlap threshold of 20%. Average number of hits per species was obtained by calculating, for each human protein, the number of hits in each group (prokaryotes and eukaryotes) divided by the number of genomes searched in each group. The average number of hits in prokaryotic species was then divided by the average number of hits in eukaryotic species to obtain ‘average ratios’. Permutation tests were done by generating 5000 groups of 2218 proteins randomly selected from the whole human proteome. The average ratios for each group of proteins was calculated and their distribution compared to the average ratios obtained for the TAPSLIVR-set (Figure 10A), obtaining the z-score which was then used to calculate the *P*-value.

Protein sequences homologous to the TAPSLIVR-set were screened for the presence of low-complexity TAPSLIVR-rich regions as described above (see 'In silico detection of low-complexity of TAPSLIVR-rich genes'). Protein sequences containing TAPSLIVR-rich regions were used to generate the heatmap shown in Figure 10B. For data normalization, species were allocated to different taxonomic groups according to their phylum, and the number of species with at least one homolog sequence was divided by the number of species in the phylum. Species were also grouped based on whether they are unicellular or multicellular. R v3.5.3 was used to generate Boxplots (package ggplot2 v3.3.2) and calculate statistical significances the Mann-Whitney *U* test.

### Analyses of A34-tRNA gene content

Total tRNA gene content for each species was obtained from the Genomic tRNA database v2.0 (58) and from (10), acquiring information for 168 of the eukaryotic species used for homology search (see above). The relationship between A34-tRNA gene content and abundance of found TAPSLIVR-rich homologs per species was evaluated by a Spearman's rank correlation coefficient using R v3.5.3 and the package ggpubr v0.2.5. For correlation tests reported in Figure 10C, seven species with an unusual number of A34-tRNA genes (>400 genes) were excluded from the analyses and are reported in Supplementary Table S6. Of note, the correlation strength reported in Figure 10C (*R* = 0.53; *P*-value = 3.7e–13) was maintained when the seven excluded eukaryotic species were included in the analyses (*R* = 0.53, *P*-value = 1e–13). For results depicted in Supplementary Figure S7, 1324 species from Bacteria and 114 species from Archaea were analysed (Supplementary Table S6).

### Statistical analyses

Statistical analyses were performed with GraphPad Prism software v6.0 and R v3.5.3. Unless stated otherwise, data shows mean ± SD of at least three biological replicates. Statistical significance was obtained by a two-tailed *t* test (*P*-value < 0.05). For RNA-Seq and tRNA-Seq data, statistical significance was defined by Benjamini-Hochberg adjusted *P*-values (adj *P*-value < 0.1, and adj *P*-value < 0.05, respectively). For whole proteomics analyses (iTRAQ), statistical significance was defined by Benjamini-Hochberg adj *P*-value < 0.1). For the analyses of homolog proteins, statistical significance when comparing average ratios was obtained using the *z*-score. Correlation analyses were performed with a Spearman's rank correlation coefficient. Statistical significance for Figure 10E was obtained by Mann–Whitney *U* test. Statistical significance of the enrichment for candidate genes among gene lists was done via Fisher Exact Test and permutation analysis.

## RESULTS

### ADAT2 KD reduces I34 levels without affecting general protein synthesis

To study the biological relevance of I34-tRNAs in HEK293T cells we used CRISPR/Cas9 technology to disrupt the *ADAT2* or *ADAT3* genes. Both genes are essential in all model organisms studied to date, and eukaryotic I34-tRNAs are absolutely required to translate C-ended codons for TAPSLIVR in species that lack G34-tRNA isoacceptors (6,14,16,17,19,20) (see also Introduction). Thus, as expected, we were unable to obtain full ADAT2 or ADAT3 knockout clones (see Materials and methods), but we did obtain heterozygous clones carrying wild type (WT) and edited alleles (‘HEK293T ADAT2 KD’ or ‘HEK293T ADAT3 KD’) (Supplementary Figure S1). Editing of the ADAT2 allele resulted in the generation of a premature stop codon eight amino acids downstream of the edited site (Supplementary Figure S1A); while editing of the ADAT3 allele resulted in the elimination of seven residues mapping to the deaminase domain of the protein without changes in the translation reading frame (Supplementary Figure S1B).

**Figure 2.**
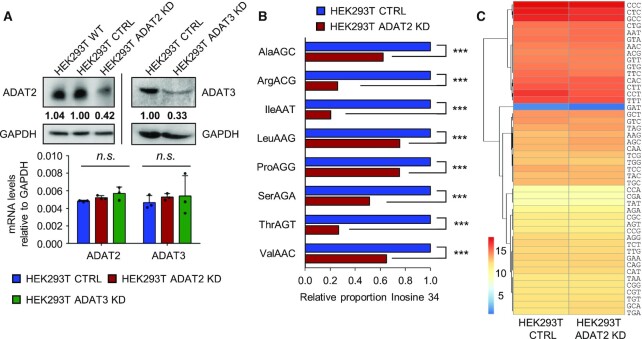
(**A**) Upper panel: ADAT2 and ADAT3 protein levels. Quantification of gel bands have been normalized to GAPDH and relative to HEK293T CTRL cells. Lower panel: ADAT2 and ADAT3 transcript levels in the indicated cell lines, relative to GAPDH. Shown are biological triplicates, their mean and standard deviations (SD). n.s.: not statistically significant (*t*-test). See also Supplementary Figure S2A. (**B**) I34 levels in HEK293T CTRL (blue) and ADAT2 KD (red) cells as evaluated by tRNA-Seq. Shown are I34 proportions relative to CTRL cells of two biological replicates calculated as in (17). ***: adj. *P*-value < 0.001 (Benjamini–Hochberg, Fisher Exact Test). See also Supplementary Figure S2B. (**C**) Heatmap visualization of tRNA gene expression at isodecoder level (tRNAs with the same anticodon) in HEK293T CTRL and ADAT2 KD cells as evaluated by tRNA-Seq. Colouring scale represents log2 DESeq2 normalized expression values based on two biological replicates calculated as in (51). No statistically significant differences were found (Benjamini–Hochberg, Fisher Exact Test, adj. *P*-value < 0.05). See also Supplementary Figure S2C and Supplementary Table S2.

Both KD cell lines presented reduced levels of the protein coded by the targeted gene (Figure 2A). ADAT2 KD did not affect the levels of ADAT3, but we observed a mild decrease in ADAT2 protein abundance upon ADAT3 KD (Supplementary Figure S2A). We did not detect changes in ADAT2 or ADAT3 mRNA levels in either cell line (Figure 2A). This is consistent with the effects of CRISPR/Cas9 targeting, and suggests that the artificially edited ADAT2 transcript with a premature stop codon can escape the nonsense-mediated decay pathway (66). HEK293T ADAT2 KD cells were stable in culture, but HEK293T ADAT3 KD cells rapidly reverted to the WT sequence (not shown).

Upon ADAT2 KD, we detected reduced levels of I34 on all its tRNA substrates, as seen by next generation sequencing of tRNAs (Figure 2B), without significant variations in tRNA transcript abundance (Figure 2C and Supplementary Table S2). As a control, we verified that the amount of the unrelated tRNA modification 1-methylinosine (m^1^I) present at position 37 of tRNA^Ala^, and catalysed by ADAT1 (67), was not affected (Supplementary Figure S2B). Similar results were observed upon shRNA-mediated KD of ADAT2 (17) (Supplementary Figure S2B-C). Because complete depletion of I34 is not possible, our cellular models allow us to identify cellular processes most sensitive to a reduction of I34-tRNAs.

**Figure 3.**
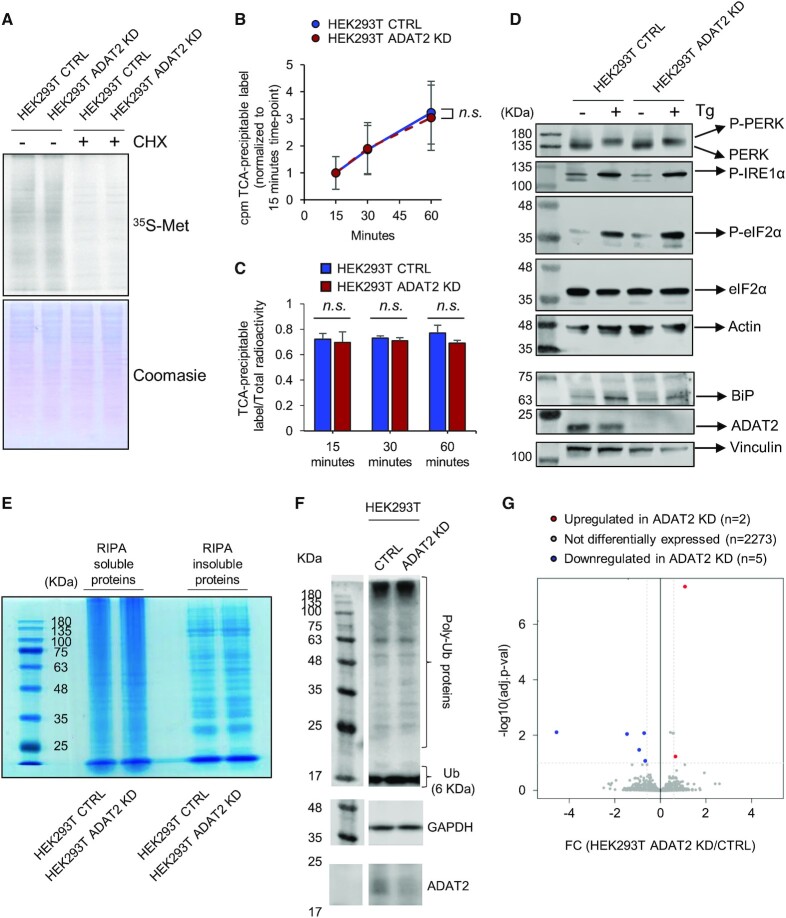
(**A**) Pulse-chase analyses on HEK293T CTRL and ADAT2 KD cells in the presence (+) or absence (−) of cycloheximide (CHX), that inhibits cytosolic translation. Coomassie staining is used as gel loading control. See also Supplementary Figure S2D. (**B**) Kinetics of protein synthesis in HEK293T CTRL (blue) and ADAT2 KD (red) cells upon monitoring the amount of TCA-precipitable label (^35^S-Met/Cys) from each cell lysate at the indicated time-points. Shown are the mean and standard deviations from biological triplicates. n.s.: not statistically significant (*t*-test) (**C**) Quantitative evaluation of ^35^S-Met/Cys incorporation into proteins upon monitoring the ratio between the total radioactivity (cpm) of the cell lysate and the amount of radioactivity (cpm) in TCA-precipitable fractions from samples in (B). Shown are the mean and standard deviations from biological triplicates. n.s.: not statistically significant (*t*-test). See also Supplementary Figure S2E. (**D**) Analyses of UPR markers on the indicated cell lines in the presence (+) or absence (−) of thapsigargin (Tg), that induces UPR. ADAT2 levels are shown for reference. Actin and Vinculin are used as gel loading controls. (**E**) Evaluation of RIPA-soluble and RIPA-insoluble protein abundance in HEK293T CTRL and ADAT2 KD cells by BlueSafe staining. (**F**) Evaluation of ubiquitination levels in total protein lysates from HEK293T CTRL and ADAT2 KD cell lines. Note that under these conditions free ubiquitin (6 KDa) runs with the front dye (∼ 17 KDa). GAPDH is used as gel loading control and ADAT2 levels are shown for reference. (**G**) Volcano plot showing differentially expressed proteins (|FC| > 1.5; adj. *P*-value < 0.1) in ADAT2 KD cells as compared to CTRL cells based on whole proteomics analysis by Mass Spec (iTRAQ). See also Supplementary Table S3.

Reduced levels of I34-tRNAs would be expected to impair cellular translation. However, pulse-chase analyses of general protein synthesis did not reveal defects in overall translation efficiency in ADAT2 KD and shADAT2 cells (Figure 3A and Supplementary Figure S2D). Quantitative metabolic labeling demonstrated that the amount of synthesized protein over time is similar in CTRL and ADAT2 KD cells (Figure 3B), and that the incorporation of free radiolabeled amino acid into proteins occurs at similar rates in both cell lines (Figure 3C). As a control, cycloheximide (CHX) treatment abolished the incorporation of radiolabeled amino acid into proteins in both cell lines (Supplementary Figure S2E).

As a proxy for studying mistranslation, we monitored activation of the unfolded protein response (UPR) (68), formation of RIPA-insoluble protein aggregates (69), and ubiquitination levels in whole protein extracts (70). Based on these parameters, we were unable to detect signs of mistranslation in ADAT2 KD cells (Figure 3D-F). We also performed mass spectrometry-based whole proteomics analyses (iTRAQ) and found only 7 differentially expressed proteins (|FC| > 1.5; adj. *P*-value < 0.1) among 2280 detected proteins (Figure 3G and Supplementary Table S3), indicating that 99.7% of detected proteins present unaltered levels under these conditions. Mass spectrometry data also showed that all detected peptides presented their expected mass for identification in all samples, indicating lack of mistranslation. Thus, depletion in I34-tRNAs caused by the inactivation of a single *ADAT2* allele (or by shRNA-mediated KD) does not cause appreciable defects in global translation efficiency or accuracy.

### Reduced I34 levels affect cell growth, and cause morphology defects

**Figure 4.**
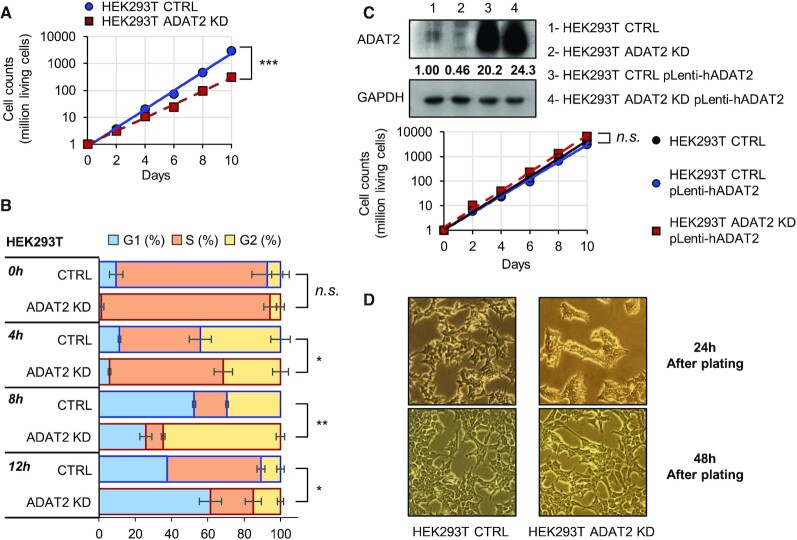
(**A**) Growth curves of HEK293T CTRL (blue) and ADAT2 KD (red) cells showing total living cells over time. Y-axis is set in logarithmic scale and an exponential trendline was fit to the data points. Data points correspond to the mean and SD of three biological replicates. ***: *P*-value < 0.001 (*t*-test). See also Supplementary Figure S3A–C. (**B**) Cell cycle analysis of HEK293T CTRL (blue-bordered bars) and ADAT2 KD (red-bordered bars) cells as evaluated by FACS over a 12-h period. Shown are the mean and SD of biological duplicates. Statistical significance is depicted only for percentage of cells in G1 phase at every time point. n.s.: not statistically significant. *: *P*-value < 0.05. **: *P*-value < 0.01 (*t*-test). (**C**) Upper panel: ADAT2 protein levels on the indicated cell lines. GAPDH is used as gel loading control. Quantification of ADAT2 bands have been normalized to GAPDH and relative to HEK293T CTRL cells. Lower panel: Growth curves as in (A) of HEK293T CTRL cells (black) and of HEK293T CTRL and ADAT2 KD cells upon stable re-expression of ADAT2 (‘pLenti-hADAT2′) (blue and red, respectively). n.s.: not statistically significant (*t*-test). (**D**) Representative light microscopy images of trypsin-treated HEK293T CTRL and ADAT2 KD cells at 24 (upper panels) and 48 (lower panels) h after plating. See also Supplementary Figure S3D.

We measured cell growth to assess the general physiological state of the cell after silencing ADAT2 or ADAT3, and found a reduced growth rate caused by a general deceleration of the cell cycle (Figure 4A, B and Supplementary Figure S3A). We observed similar phenotypes in different shADAT2 human cell lines (Supplementary Figure S3B and C), and we were able to fully recover growth rates in ADAT2 KD cells by introduction of a lentiviral ADAT2 expression system (‘HEK293T ADAT2 KD pLenti-hADAT2′) (Figure 4C). Thus, the observed phenotypes are due to reduced levels of I34-tRNAs caused by ADAT depletion. We noticed that cells depleted of I34-tRNAs presented an abnormal morphology after being detached by trypsin treatment and re-plated in clean culture plates. This phenotype was transient (Figure 4D), and absent in cells detached using PBS-EDTA (Supplementary Figure S3D). This suggests that the silencing of ADAT2, and the resulting reduction in levels of I34-tRNAs, impair the ability of cells to recover from the proteolytic elimination of membrane proteins exposed to the extracellular milieu.

### Depletion of I34-tRNAs impairs cell adhesion and sensitises cells to translation inhibitors

**Figure 5.**
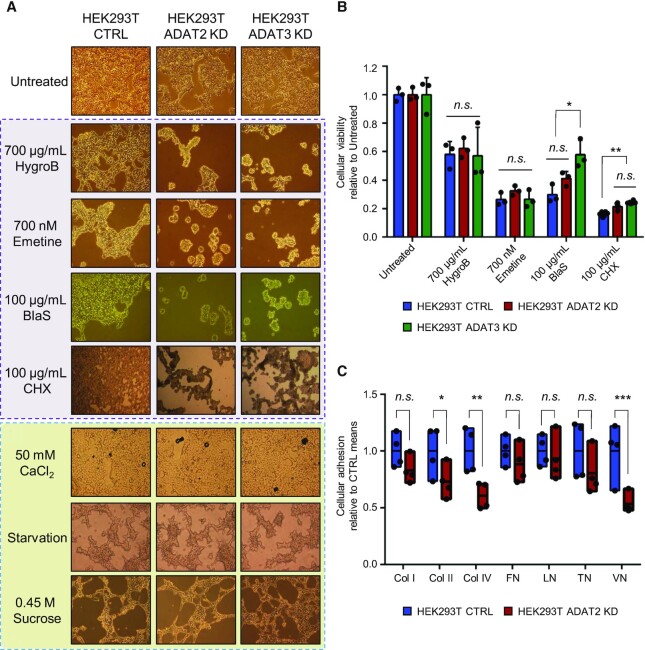
(**A**) Representative light microscopy images of HEK293T CTRL, ADAT2 KD and ADAT3 KD cells in the presence of the indicated stress agents. Those that directly affect the translation machinery are highlighted in purple. HygroB: hygromycin B. BlaS: blasticidin S. CHX: cycloheximide. (**B**) Cellular viability (metabolic activity) measured by WST-1 assays for the indicated cell lines in the presence of antibiotics depicted in (A). Shown are biological triplicates, their means and SD relative to untreated cells. n.s.: not statistically significant. *: *P*-value < 0.05. **: *P*-value < 0.01 (*t*-test). (**C**) Evaluation of cell adhesion to components of the extracellular matrix. Col I, II and IV: collagen I, II and IV respectively. FN: fibronectin. LN: laminin. TN: tenascin. VN: vitronectin. Box plots represent min-to-max cell adhesion relative to the means of HEK293T CTRL cells based on four biological replicates. n.s.: not statistically significant. *: *P*-value < 0.05. **: *P*-value < 0.01. ***: *P*-value < 0.001 (*t*-test). See also Supplementary Figure S3E.

We then tested whether translation machinery inhibitors would have a synergistic effect with ADAT silencing. We found that both ADAT2 KD and ADAT3 KD cells, but not CTRL cells, spontaneously detached from culture plates upon treatment with antibiotics such as Hygromycin B (HygroB), Emetine, Blasticidin S (BlaS) and Cycloheximide (CHX) (Figure 5A). In contrast, all three cell lines remained adhered to culture plates when exposed to insults that do not directly affect translation, such as calcium chloride (CaCl_2_), starvation, or incubation in hyperosmotic media (0.45 M Sucrose) (Figure 5A). We further found that detached cells treated with antibiotics were viable, grew normally if re-plated in clean culture plates (not shown), and were metabolically equal to CTRL cells (Figure 5B), indicating that their detachment was not due to a differential sensitivity to antibiotic toxicity. Thus, although our data shows that global translation is not affected in cells with reduced I34-tRNAs, we observe phenotypes consistent with impaired translation of specific functional protein families.

These results prompted us to investigate whether I34-tRNA depletion quantitatively impairs the adhesion capacity of cells. Furthermore, because cellular morphology and proliferation depends upon cellular adhesion (71,72), compromised cell adhesion can also explain the phenotypes observed in trypsin-treated ADAT2 KD cells (Figure 4). We reasoned that the observed phenotypes could be caused by impairment in the *de novo* synthesis of membrane proteins necessary for cell attachment. To evaluate the adhesion capacity of I34-depleted cells in a context where *de novo* translation is required for this function we: (i) treated ADAT2 KD and CTRL cells with trypsin to degrade plasma membrane proteins and stimulate their synthesis; (ii) plated the cells in standard culture plates for 24 h; (iii) harvested the cells with PBS-EDTA (preserving all newly synthesized membrane proteins) and (iv) placed the cells in plates previously coated with individual components of the extracellular cell matrix (ECM) to test the ability of the cells to bind to physiological substrates. We found that ADAT2 KD cells display impaired adhesion to collagens (Col II, Col IV) and vitronectin (VN), but not to fibronectin (FN), laminin (LN) or tenascin (TN) (Figure 5C and Supplementary Figure S3E). These results indicate that upon degradation of membrane proteins cells depleted from I34-tRNAs fail to efficiently resynthesize proteins required for cellular attachment to specific components of the ECM.

### Depletion of I34-tRNAs reduces ribosome occupancy on a subset of transcripts

**Figure 6.**
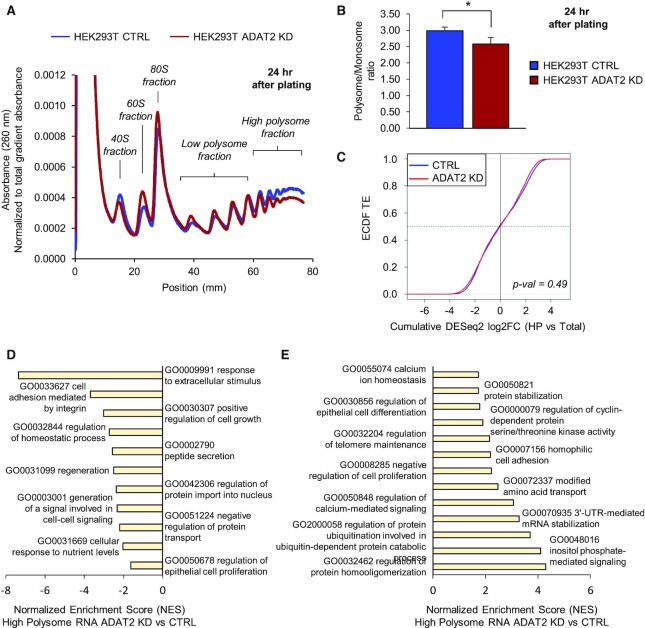
(**A**) Representative polysome profile of trypsin-treated HEK293T CTRL (blue) and ADAT2 KD (red) cells at 24 h after plating. The 40S, 60S, 80S, Low polysome, and High polysome (HP) fractions are indicated for reference. Experiments were done in biological triplicates. See also Supplementary Figure S4A. (**B**) Polysome to Monosome ratio (P/M) obtained from experiments as in (A). Shown are the mean and standard deviations of biological triplicates. *: *P*-value < 0.05 (*t*-test). (**C**) Evaluation of translation efficiency (TE) of transcripts based on their expression in total RNA (Total) and high polysome fraction (HP). Shown are the Empirical Cumulative Distribution Function (ecdf) of translation efficiencies for HEK293T CTRL (blue) and HEK293T ADAT2 KD (red) cells. Statistical test was performed using the mded function to compare empirical distributions (57). (**D** and **E**) ROAST Gene Set Enrichment Analysis using the Gene Ontology set Biological Process for transcripts depleted (D) or enriched (E) in the HP fraction of HEK293T ADAT2 KD cells. Selected categories are shown. Adj. *P-*value < 0.05. See also Supplementary Table S4.

To explore the impact of I34-tRNAs on the translatome we performed polysome profiling at 24 h after trypsin treatment and plating. We detected reduced levels of mRNAs in the high polysomal fractions and a consequent increase of mRNA abundance present in the low polysomal fractions in ADAT2 KD cells (Figure 6A), indicating reduced ribosome occupancy on transcripts. In addition, we found an increase in the 80S ribosomal fraction and a shift in the 40S-to-60S ribosomal fraction ratio (Figure 6A). At 72 h after trypsin treatment and plating, we observed these differences substantially reduced, consistent with proteome normalization after protease treatment (Supplementary Figure S4A).

To quantitatively assess these differences, we calculated polysome to monosome ratios (P/M ratio). We found that ADAT2 KD cells presented a significant depletion in the P/M ratio at 24 h after trypsin treatment (Figure 6B), a difference that disappeared at 72 h after treatment (Supplementary Figure S4B). This data is consistent with the hypothesis that the modest effects on ribosome occupancy observed are due to translation impairment of a subset of genes, while global translation is generally not affected.

To characterize transcripts differentially translated in ADAT2 KD cells, we performed RNA-Seq at 24 h after trypsin treatment and plating, both from input RNA (‘Total RNA’ to assess transcriptomic changes) as well as from RNA obtained from the high polysome (HP) fractions. We detected a significant depletion of ADAT2 transcripts (FC < 1.5; adj. *P*-value < 0.1) in the HP fraction of the ADAT2 KD cell line without significant changes in total RNA (Supplementary Figure S4C), indicating translational impairment. This is consistent with ribosomal drop-off caused by the premature stop codon introduced in this gene by CRISPR/Cas9 editing (Supplementary Figure S1A, see also Figure 2A). In agreement with previous observations, we did not observe alterations of ADAT3 transcript levels in total RNA or HP fractions in this cell line (Supplementary Figure S4C, see also Figure 2A and Supplementary Figure S2A).

Although we found 726 differentially enriched or depleted (|FC| > 1.5; adj. *P*-value < 0.1) protein-coding genes in the HP fractions of ADAT2 KD cells (Supplementary Table S4), a global analysis of translation efficiency (TE) (cumulative log_2_FC HP versus total) found no major differences in ADAT2 KD cells compared to CTRL cells (*P*-value = 0.49; Figure 6C). This is in agreement with our previous observations that general translation is not affected in ADAT2 KD cells, and indicates that most of the differential expression observed in HP fractions can be explained by changes in transcriptional rates.

Despite the fact that general translation efficiency is not affected by depletion of I34-tRNAs, gene ontology (GO) analyses revealed compositional differences in the HP fractions of ADAT2 KD cells. Indeed, HP fractions after ADAT2 KD are significantly depleted in transcripts associated to cellular proliferation, cell adhesion, cell-cell signalling, response to extracellular stimuli, protein transport and peptide secretion functions. On the other hand, HP fractions after ADAT2 KD are significantly enriched in transcripts linked to cellular differentiation, calcium signalling, protein and mRNA stabilization, telomere maintenance, and protein ubiquitination (Figure 6D, E and Supplementary Table S4). Thus, the depletion of I34-tRNAs does not affect general translation efficiency, but induces changes in the composition of transcript populations associated to ribosomes.

### Impact of I34-tRNA depletion upon translation depends on codon composition and distribution

**Figure 7.**
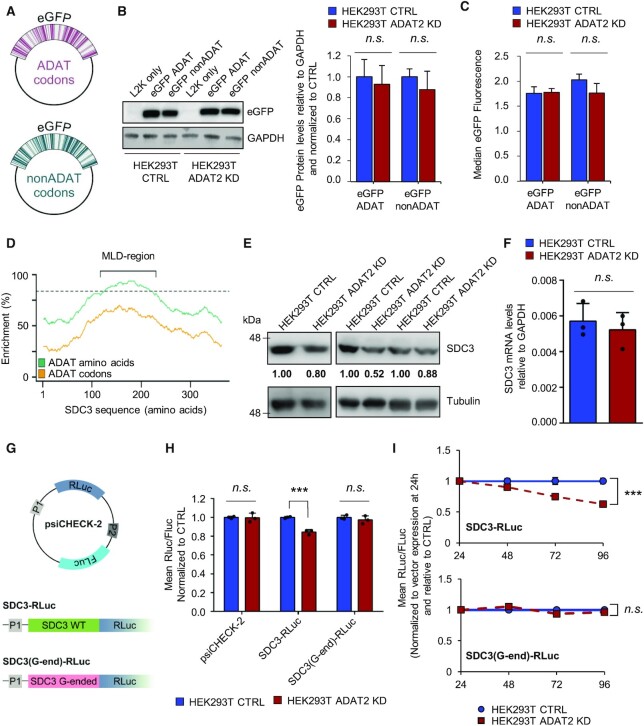
(**A**) Schematic representation of constructs encoding eGFP with TAPSLIVR codons that are C-ended (ADAT codons, upper panel in violet) or G-ended (nonADAT codons, lower panel in dark green). (**B**) eGFP protein levels in the indicated cell lines when transfected with eGFP constructs depicted in (A). GAPDH is used as gel loading control. ‘L2K only’: cells incubated with lipofectamine 2000 in the absence of eGFP constructs. Quantification of western blot bands for eGFP relative to GAPDH levels and normalized to CTRL cells was obtained from three biological replicates. Shown are the mean and SD. *n*.s.: not statistically significant (t-test). See also Supplementary Figure S5A–C. (**C**) Evaluation of eGFP fluorescence by FACS in the indicated cell lines when transfected with eGFP constructs depicted in (A). Shown is the median and SD of biological duplicates. n.s.: not statistically significant (*t*-test). (**D**) Distribution of TAPSLIVR (ADAT amino acids; green curve) and of codons decoded by I34-tRNAs (ADAT codons; orange curve) along the coding sequence of SDC3. Dotted line indicates the threshold of TAPSLIVR enrichment considered significant as defined in (23). The low-complexity TAPSLIVR-rich region containing the Mucin-like domain (MLD) is shown. (**E**) SDC3 protein levels in HEK293T CTRL and ADAT2 KD cells. Tubulin is used as gel loading control. Quantification of SDC3 bands relative to Tubulin and normalized to CTRL cells for each biological triplicate is shown. (**F**) SDC3 transcript levels relative to GAPDH in the indicated cell lines. Shown are biological triplicates, their mean and SD. n.s.: not statistically significant (*t*-test). (**G**) Schematic representation of psiCHECK-2-based SDC3 luciferase reporters bearing the wild-type low-complexity region of SDC3 (‘SDC3 WT’ in light green) or the same amino acid sequence but encoded with synonymous G-ended codons for all TAPSLIVR (codons not recognised by I34-tRNAs, ‘SDC3 G-ended’ in pink). The promoter 1 (P1) drives the expression of Renilla luciferase (RLuc). The second independent promoter (P2) drives the expression of Firefly luciferase (FLuc). (**H**) Evaluation of luciferase expression (RLuc/FLuc ratio normalized to CTRL cells) in HEK293T CTRL and ADAT2 KD cells transfected with the constructs depicted in (G). Shown are biological triplicates, their mean and SD. n.s.: not statistically significant. **: *P*-value < 0.01 (*t*-test). (**I**) Time-course analysis of luciferase expression (RLuc/FLuc ratio normalized to CTRL cells and to vector expression at the 24 h time-point) in the indicated cell lines transfected with SDC3-RLuc (upper panel) or SDC3(G-end)-RLuc (lower panel). Shown are the means (blue for CTRL and red for ADAT2 KD cells) and SD of biological triplicates. n.s.: not statistically significant. ***: *P*-value < 0.001 (*t*-test). See also Supplementary Figure S5D.

To gauge the relationship between codon composition and I34-tRNA dependence, we first assessed the impact of ADAT depletion upon translation of proteins with an even distribution of TAPSLIVR in their sequences. To that end we engineered two eGFP genes where codons for TAPSLIVR were either C-ended (ADAT-sensitive; ‘eGFP ADAT’), or G-ended (ADAT-insensitive; ‘eGFP nonADAT’) (Figure 7A, see also Materials and methods). GFP reporters are frequently used for the analysis of codon-biased translation (73). Importantly, to prevent differences in translation rates caused simply by the changes in codon usage, we ensured that these two eGFP sequences would share a similar Codon Adaptation Index (CAI) (74).

We observed similar levels of total eGFP protein and fluorescence in ADAT2 KD and CTRL cells when transfected with either eGFP variant by western blotting and FACS analyses (Figure 7B, C, respectively). This indicates that both eGFP variants are translated at a similar rate and that they fold into their active form to produce fluorescence in both cell lines. Likewise, we detected equivalent eGFP production in HEK293T shCV and shADAT2 cells, and for both expression constructs (Supplementary Figure S5A and B). Thus, eGFP translation is not sensitive to partial I34-tRNA depletion, even if codon composition is maximally biased towards I34-tRNA use. In addition, we did not find signs of mistranslation based on peptide analysis by mass spectrometry (Supplementary Figure S5C). Therefore, a reduction in I34 levels had no effect upon the efficiency or the fidelity of translation of a soluble protein containing evenly distributed TAPSLIVR. This is consistent with the observation that translation of soluble proteins of average amino acid composition remains unaffected upon ADAT2 KD (Figure 3).

We have previously shown that the frequency of codons recognised by I34-tRNAs in eukaryotic genes positively correlates with the number of consecutive TAPSLIVR-encoding codons in the corresponding proteins (10,23,24). We therefore asked whether I34 levels are important for the synthesis of proteins with low-complexity TAPSLIVR-rich regions. First, we identified human transcripts encoding proteins with low-complexity TAPSLIVR-rich regions, and ranked them according to the size of these regions, and their relative enrichment in TAPSLIVR codons cognate for I34-tRNAs (Supplementary Table S5). Next, we performed an *in silico* functional characterization of the identified low-complexity TAPSLIVR-rich proteins. We found that this subset of the human proteome is associated to cellular structure, morphology, adhesion, cell signalling, and interaction with the extracellular space (Supplementary Table S5). We further found that these low-complexity regions are characteristic of mucin-like domains (MLDs) (75), and are abundant in Mucins (MUC) and other proteins involved in ECM regulation and adhesion (Supplementary Table S5).

We monitored endogenous levels of the MLD-containing protein Syndecan 3 (gene *SDC3*) (76) (Figure 7D), as a function of ADAT2 levels. We found that cells depleted of I34-tRNAs produce less SDC3 compared to CTRL cells, without significant changes in SDC3 transcript abundance (Figure 7E and F). To test if this effect was due to translation impairment of the low-complexity MLD region of SDC3 we generated a reporter gene where this section of the SDC3 transcript (Figure 7D) was cloned at the N-terminus of a Renilla luciferase (RLuc) gene (SDC3-RLuc). We also generated an equivalent construct (SDC3(G-end)-RLuc) where all ADAT-sensitive codons (U-, C- and A-ended codons) of the cloned region of SDC3 were replaced by G-ended codons, thus rendering them I34-tRNA-insensitive (decoded by C34-tRNAs) (see Materials and methods). Both constructs contain a Firefly luciferase (FLuc) that acts as an internal control for normalization of expression (Figure 7G).

We found a 20% reduction in SDC3-RLuc expression, but not of SDC3(G-end)-RLuc, in ADAT2 KD cells at 48 h after transfection (Figure 7H). A time-course analysis revealed a continued decrease of SDC3-RLuc in ADAT2 KD cells relative to CTRL cells (Figure 7I). We purified SDC3-RLuc and SDC3(G-end)-RLuc from all cell lines and found their protein sequences to be identical by mass spectrometry (Supplementary Figure S5D). Thus, in contrast to transcripts with evenly distributed TAPSLIVR codons, a partial depletion of I34-tRNAs impairs the translation of low-complexity TAPSLIVR-rich transcripts.

**Figure 8.**
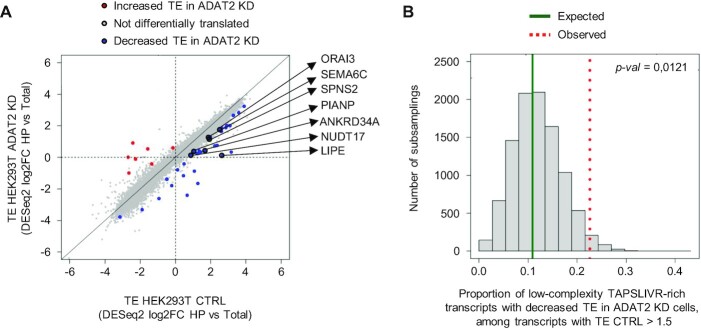
(**A**) Scatter plot showing the evaluation of translation efficiency (TE) for individual transcripts based on an interaction analysis between transcript expression in total RNA (Total) and high polysome (HP) fractions obtained for experiments as in Figure 6. Blue, Red and Grey dots indicate genes with decreased TE, increased TE and unchanged TE in HEK293T ADAT2 KD cells as compared to HEK293T CTRL cells, respectively. Note that the x- and y-axis show Log2FC values (log2FC 1.5 = 0.58). Translationally impaired transcripts that encode proteins with low-complexity TAPSLIVR-rich regions are indicated. See also Supplementary Table S4. (**B**) Histogram of a permutation test using 10 000 sets of 31 random transcripts detected in experiments shown in (A). The expected (dark solid green line) and observed (red dotted line) proportion of transcripts encoding low-complexity TAPSLIVR-rich proteins with decreased TE in ADAT2 KD cells among transcripts highly translated in HEK293T CTRL cells (FC HP CTRL versus Total CTRL > 1.5) is indicated. *P*-values are computed as the proportion of permutations with more extreme statistics than the observed. See also Supplementary Table S4.

In light of this evidence we revisited our polysome profiling data to evaluate the specific synthesis of low-complexity TAPSLIVR-rich proteins in ADAT2 KD cells. Using an interaction analysis (see Materials and methods) we found that 7 out of the 36 genes with impaired TE in ADAT2 KD cells (FC HP versus total < 1.5; *P*-value < 0.05) encoded proteins with TAPSLIVR-rich low-complexity regions (Figure 8A and Supplementary Table S4). Notably, we found that under these conditions, these transcripts are highly translated in CTRL cells (i.e. FC HP CTRL versus total CTRL > 1.5; *P*-value < 0.05) (Figure 8A). A permutation test revealed that the fraction of translationally impaired transcripts in ADAT2 KD cells that are highly translated in CTRL cells (31 genes) is enriched in low-complexity TAPSLIVR-rich coding sequences (10 000 sets of 31 random genes detected in the polysome profiling experiment, *P*-value = 0.0121; Figure 8B). These results suggest that ADAT2 KD causes impaired translation of transcripts that require I34-tRNAs and are under high translational demand.

To extend this analysis to a larger set of transcripts we relaxed the statistical constraints imposed on the abovementioned interaction analysis, and evaluated the TE (i.e. upregulated (FC > 0) or downregulated (FC < 0)) upon ADAT2 KD without setting up a FC or *P*-value threshold. We found 989 transcripts encoding proteins with low-complexity TAPSLIVR-rich regions with downregulated TE, representing a statistically significant enrichment among all detected transcripts with downregulated TE (*P*-value = 0.03036; Fisher exact test). This significance is increased when the analysis is restricted to highly translated transcripts in CTRL cells (i.e. FC HP CTRL versus Total CTRL > 0) (550 transcripts encoding low-complexity TAPSLIVR regions; *P*-value = 4.985e–14; Fisher exact test) (Supplementary Table S4). These analyses support the observation that transcripts encoding proteins with low-complexity TAPSLIVR-rich regions are enriched among those translationally impaired upon ADAT2 KD, particularly if such transcripts are under high translational demand.

### Depletion of I34-tRNAs impairs translation of MLD-containing proteins in different cell lines

**Figure 9.**
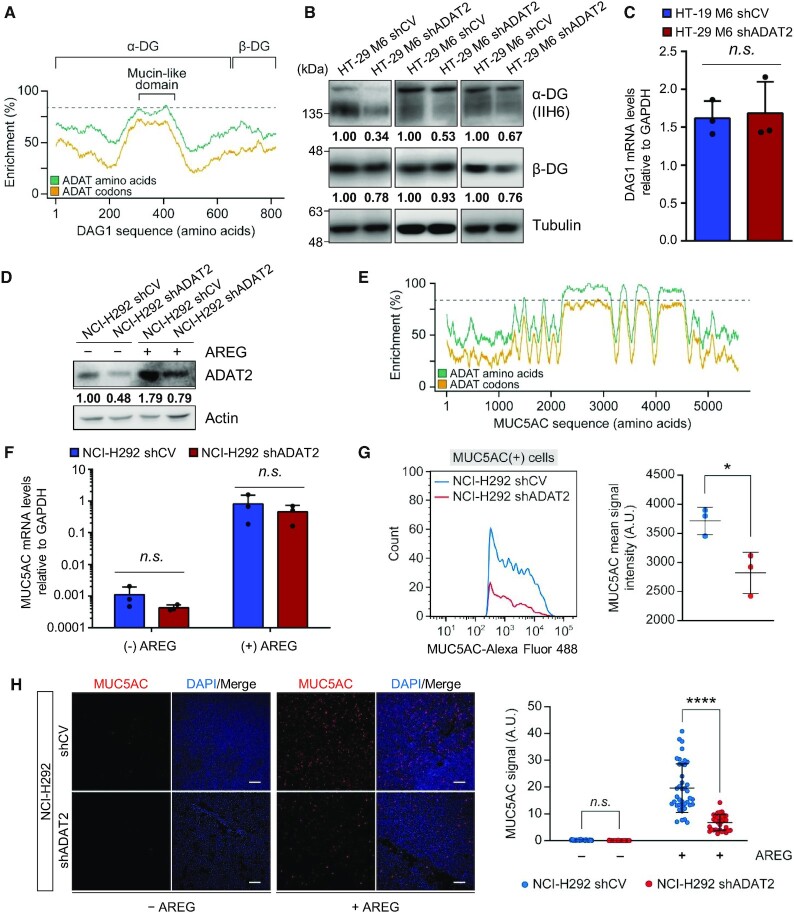
(**A**) Distribution of TAPSLIVR (ADAT amino acids; green curve) and of codons decoded by I34-tRNAs (ADAT codons; orange curve) along the coding sequence of DAG1. Dotted line indicates the threshold of TAPSLIVR enrichment considered significant as defined in (23). The low-complexity TAPSLIVR-rich region containing the Mucin-like domain, and the α and β dystroglycan (DG) subunits are shown. (**B**) α-DG and β-DG protein levels in HT-29 M6 shCV and shADAT2 cells. Tubulin is used as gel loading control. Quantification of DG bands relative to Tubulin and normalized to shCV cells for each biological triplicate is shown. Note that for each replicate α-DG and β-DG were measured from the same protein extract. ADAT2 levels in each protein extract is shown in Supplementary Figure S6A. (**C**) DAG1 transcript levels relative to GAPDH in the indicated cell lines. Shown are biological triplicates, their mean and SD. n.s.: not statistically significant (*t*-test). (**D**) ADAT2 protein levels in NCI-H292 shCV and shADAT2 cells treated (+) or not (−) with amphiregulin (AREG). Actin is used as gel loading control. Quantification of ADAT2 bands relative to Actin and normalized to shCV cells (−) AREG is shown. See also Supplementary Figure S6B-C. (**E**) Distribution of TAPSLIVR (ADAT amino acids; green curve) and of codons decoded by I34-tRNAs (ADAT codons; orange curve) along the coding sequence of MUC5AC. Dotted line indicates the threshold of TAPSLIVR enrichment considered significant as defined in (23). (**F**) MUC5AC transcript levels relative to GAPDH in the indicated cell lines treated (+) or not (−) with AREG. Shown are biological triplicates, their mean and SD. n.s.: not statistically significant (*t*-test). (**G**) MUC5AC expression levels upon AREG treatment in NCI-H292 shCV (blue) or shADAT2 (red) cells, quantified by FACS. Only MUC5AC(+) cells are shown. Left panel: representative assay showing number of MUC5AC(+) cells (y-axis) and MUC5AC signal intensity (x-axis). Right panel: MUC5AC mean signal intensity obtained for three biological replicates. The mean of the means and SD is also shown. *: *P*-value < 0.05 (*t*-test). See also Supplementary Figure S6D and E. (**H**) MUC5AC expression in NCI-H292 shCV and shADAT2 cells treated (+) or not (−) with AREG, quantified by confocal microscopy. Left panel: representative confocal microscopy images. MUC5AC (red) and nuclei staining (DAPI, blue) are shown. Scale bar corresponds to 10 μm. Right panel: quantification of microscopy images (*n* > 30) as those shown in Left panel for the indicated cell lines with (+) or without (−) AREG treatment with their mean and SD. n.s.: not statistically significant. ****: *P*-value < 0.0001 (*t*-test).

To rule out cell-specific effects we evaluated the endogenous levels of two additional low-complexity MLD-containing proteins in different cellular model systems. First, we examined the expression of Dystroglycan 1 (coded by the gene *DAG1*) (Figure 9A) in HT29-M6 shCV and shADAT2 cells (Supplementary Figure S3B, C and Supplementary Figure S6A). Dystroglycan 1 is translated from a single transcript as a propeptide that is post-translationally cleaved into two subunits: alpha-dystroglycan (α-DG) that has a low-complexity TAPSLIVR-rich MLD, and beta-dystroglycan (β-DG) (77) (Figure 9A). Therefore, defects in translation of α-DG should also impact translation of β-DG. We found that ADAT2 KD reduced the levels of both proteins (Figure 9B) without affecting DAG1 mRNA abundance (Figure 9C).

To investigate translation of mucins (MUC) in ADAT-silenced cells, we used a line of human pulmonary mucoepidermoid carcinoma cells (NCI-H292) where MUC production is induced by the epidermal growth factor-like protein amphiregulin (AREG) (78). Interestingly, AREG treatment induced ADAT2 expression in both NCI-H292 shCV and shADAT2 cells, although the latter continued to present reduced ADAT2 abundance compared to shCV cells (Figure 9D). This is consistent with the notion that ADAT activity is linked to the efficient synthesis of mucins. We evaluated a number of molecular markers of AREG-induced signalling and found that ADAT2 depletion did not generally affect the cellular response to AREG treatment (Supplementary Figure S6B-C).

We next examined the expression of mucin-5 Subtype AC (*MUC5AC*) (Figure 9E). As expected, AREG treatment sharply increased the levels of MUC5AC mRNAs (78). This activation was of ∼ 1000-fold and similar for shCV and shADAT2 cells (Figure 9F). However, we detected a strong reduction in MUC5AC protein levels in shADAT2 cells, both by FACS analyses (∼ 30% reduction, Figure 9G and Supplementary Figure S6D-E) and immunohistochemistry (∼70% reduction, Figure 9H), consistent with a severe translational defect.

### Low-complexity TAPSLIVR-rich proteins are primarily Eukarya-specific and enriched in multicellular organisms

The enrichment of I34-tRNAs in Eukarya (4,10,21), and the fact that MLDs are found mostly in eukaryotes (75), prompted us to ask whether low-complexity TAPSLIVR-rich proteins are overrepresented in Eukaryotes. Using established methods (79) we searched for homologous sequences to these human TAPSLIVR-rich proteins in all three domains of life. Evaluating homology on the basis of low-complexity regions is subject to numerous biases (80), thus we first identified homologs using the full sequence of human proteins containing TAPSLIVR-rich regions. In this way we were able to identify all proteins evolutionary related to the human query set, independently of their low-complexity TAPSLIVR-rich region.

**Figure 10.**
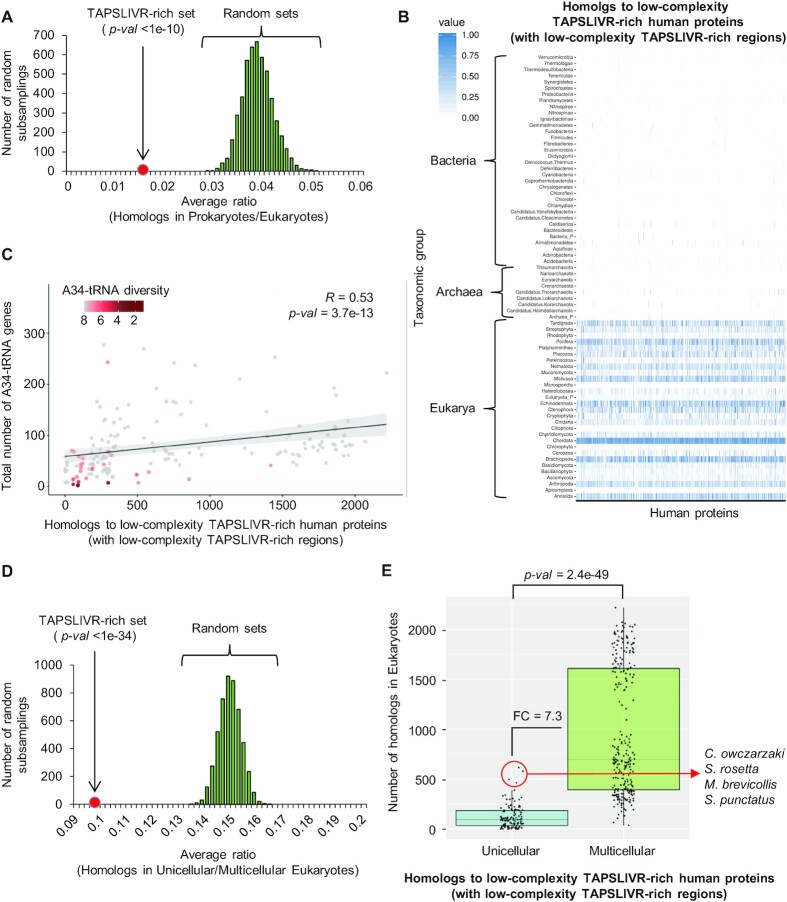
(**A**) Histogram of permutation tests using 5000 sets of random human proteins, showing the distribution of homologs in prokaryotes relative to those in eukaryotes (average ratio) for each random set analysed (subsamplings). The average ratio obtained for the human TAPSLIVR-rich set is shown (red dot). *P*-value < 1e-10 (based on z-score). (**B**) Heatmap visualization of the abundance of homologs to low-complexity TAPSLIVR-rich human proteins, and that contain low-complexity TAPSLIVR-rich regions, in taxonomic groups belonging to Archaea, Bacteria and Eukarya. Colouring scale represents hits normalized to the number of species analysed in each taxonomic group. X-axis: human proteins used as input for these analyses. (**C**) Spearman's rank correlation coefficient between the total number of A34-tRNA genes and the number of low-complexity human TAPSLIVR-rich proteins with homologs in eukaryotes. Color-coding indicates A34-tRNA diversity being 8 the maximum (i.e. at least one A34-tRNA gene to decode each TAPSLIVR). See also Supplementary Table S6 and Supplementary Figure S7. (**D**) Histogram of permutation tests using 5000 sets of random human proteins, showing the distribution of homologs in unicellular eukaryotes relative to multicellular in eukaryotes (average ratio) for each random set analysed (subsamplings). The average ratio obtained for the human TAPSLIVR-rich set is shown (red dot). *P*-value < 1e–34 (based on *z*-score). See also Supplementary Table S7. (**E**) Boxplot representation of the abundance of homologs to low-complexity TASPLIVR-rich human proteins, and that contain low-complexity TAPSLIVR-rich regions, in unicellular and multicellular eukaryotes. The identity of outlier unicellular species is shown. FC: Fold-change of the medians. Statistical significance was obtained by Mann–Whitney *U* test. See also Supplementary Table S7.

We found that the average per-species abundance of homologous sequences to low-complexity human TAPSLIVR-rich proteins is 66-fold higher in eukaryotes than in prokaryotes (homologs in prokaryotes/homologs in eukaryotes = 0.015; see Materials and methods) (Figure 10A). To evaluate the significance of this result, we performed a permutation test with 5000 sets of randomly chosen human sequences. This confirmed that low-complexity TAPSLIVR-rich protein homologs are exceedingly rare in prokaryotic organisms (*P*-value < 1e–10) (Figure 10A). We then examined the presence of low-complexity TAPSLIVR-rich regions within these proteins to find that they are almost absent in prokaryotes (Figure 10B).

Interestingly, we found an uneven distribution of homologs of these sequences within eukaryotes (Figure 10B). We asked whether this could correlate with the number of tRNA genes coding for precursors of I34-tRNAs (i.e. A34-tRNA genes) present in these species. We found a positive trend (*R* = 0.53, *P*-value = 3.7e–13, Spearman) between the abundance of low-complexity TASPLIVR-rich proteins and A34-tRNA gene content (Figure 10C and Supplementary Table S6). Furthermore, eukaryotes that lack A34-tRNA genes for any of the TAPSLIVR (i.e. reduced A34-tRNA gene diversity) are also depleted in low-complexity TAPSLIVR-rich homologs (Figure 10C). Of note and as expected, proteins with low-complexity TAPSLIVR-rich regions are rare in bacterial and archaeal genomes (Figure 10B), which are depleted of I34-tRNAs (Supplementary Figure S7).

The capacity to synthesize cell adhesion molecules was instrumental for the origin of multicellularity (81–84), and low-complexity TAPSLIVR-rich proteins are involved in cell adhesion and extracellular matrix interactions (Supplementary Table S5). We calculated the average per-species abundance of homologs to human low-complexity TAPSLIVR-rich proteins in unicellular and multicellular eukaryotes and found that they are severely depleted in unicellular species (homologs in unicellular eukaryotes/homologs in multicellular eukaryotes = 0.096; *P*-value < 1e–34 compared to 5000 sets of randomly chosen human sequences) (Figure 10D). We further evaluated the presence of low-complexity TAPSLIVR-rich regions within these proteins and detected a 7.3-fold enrichment in multicellular species (Figure 10E and Supplementary Table S7).

These results show that the scarcity or abundance of I34-tRNAs in eukaryotes correlate with the capacity of these species to synthesize proteins with low-complexity TAPSLIVR-rich regions involved in cell adhesion, and with their unicellular or multicellular condition. Interestingly, we found four unicellular eukaryotic species with an unusually high number of proteins with low-complexity TASPLIVR-rich stretches (i.e. *Capsaspora owczarzaki, Salpingoeca rosetta, Monosiga brevicollis* and *Spizellomyces punctatus*) (Figure 10E). All these species are considered model organisms to study the transition towards metazoan multicellularity (25,83–86).

## DISCUSSION

The selective pressures that drove the evolution of the translation apparatus, and their impact upon the functional and structural diversity of proteomes are unknown. More specifically, the replacement of G34-tRNAs for I34-tRNAs in eukaryotic genomes is a major event during early eukaryotic evolution that remains unexplained (4,5,21). Extant eukaryotic I34-tRNAs are essential to translate C-ended codons due to the lack of genes coding for isoacceptor G34-tRNAs (6,14,16,19,20,58). However, although this highlights an essential function of these tRNAs, it does not inform on the selective advantage that drove their expansion early in eukaryote evolution.

The expansion of I34-tRNAs during eukaryotic emergence needs to be considered in the context of the physical constraints surrounding codon-anticodon interactions. G34-tRNAs generate high-energy codon-anticodon pairings (87) which, in bacteria, require an internal base pairing between bases 32 and 38 of the anticodon loop to reduce the codon anticodon affinity through structural strains upon the loop structure (26). In eukaryotic translation systems, G34-tRNAs induce miscoding and are toxic, presumably because of non-cognate pairing of G34-tRNA anticodons with C-ended codons (26). It is conceivable that bacterial G34-tRNAs would cause a fitness conflict when used by an archaeal-type translation machinery, leading to the substitution of G34-tRNAs by an alternative tRNA. However, this scenario does not explain why I34-tRNAs would be the preferred solution to this conflict. The toxicity of G34-tRNAs in human cells could be alleviated by single base changes at positions 32 or 38 (26), moreover, I34-tRNAs may impose other constraints upon tRNA sequences. For example, eukaryotic tRNA^Ala^_AGC_ presents peculiar tertiary structures unique to this kingdom (88). Thus, additional selective forces may have contributed to the dramatic expansion of I34-tRNAs in nucleated cells.

In this work we use cellular models that are partially depleted from I34-tRNAs to levels equivalent or lower than those previously reported in human cell lines or other species upon ADAT downregulation (13,15–17,20,49,89). This depletion is achieved without causing additional alterations to the tRNA pool, and the resulting cells are still viable (Figures 2 and 4, Supplementary Figure S2B-C and Supplementary Table S2). Under these conditions, we might expect to identify cellular processes particularly sensitive to I34 depletion. We find that depletion of I34-tRNAs impairs cell adhesion (Figure 5C), and ADAT KD cells tend to detach when exposed to protein synthesis inhibitors, but not to other cellular insults (Figure 5A and B). Moreover, we observe that reduced I34 levels cause an abnormal cellular morphology upon trypsin treatments (Figure 4D and Supplementary Figure S3D), indicating that I34-tRNAs are required for the *de novo* synthesis of membrane proteins involved in interactions with the extracellular environment. We also observe reduced proliferation and a slower cell cycle (Figure 4A–C), two phenotypes commonly caused by defects in cellular adhesion (71,72) and previously reported in other cellular systems upon ADAT depletion (6,15,16,19,20). Silencing of ADAT2 also causes a notable decrease in transcripts with high ribosomal occupancy after trypsin treatment (Figure 6A), and within this group, we find an overrepresentation of genes linked to cell adhesion, response to extracellular stimuli and cell-cell signalling (Figure 6D and Supplementary Table S4), indicating that extracellular polypeptides predominate among those affected by a partial depletion of I34-tRNAs.

These observations are consistent with the hypothesis that partial I34-tRNA depletion leads to translational impairment of a specific subset of transcripts. Indeed, we do not find translation to be generally compromised when I34-tRNA levels are reduced, as shown by monitoring protein synthesis rates through metabolic labelling, analysing soluble and insoluble protein fractions, and evaluating UPR markers and levels of protein ubiquitination (Figure 3A–F and Supplementary Figure S2D). Furthermore, whole proteome mass spectrometry analyses revealed only 7 proteins out of 2280 to be differentially expressed (2 proteins upregulated and 5 downregulated) (Figure 3G and Supplementary Table S3). We favour the hypothesis that these changes are due to modulation of transcriptional rates. Likewise, no global alterations in translation efficiency were observed in ADAT2 KD cells by RNA-Seq in polysome profiling experiments (Figure 6C), and only 36 genes out of 12 447 were found to be translationally impaired (Figure 8A, and see below). This indicates that the majority of the differential abundance of transcripts associated to ribosomes found in ADAT2 KD cells could be explained by changes in transcriptional rates (Figure 6 and Supplementary Table S4).

On the other hand, we do find impaired translation of low-complexity TAPSLIVR-rich proteins that are encoded by transcripts enriched in codons cognate for I34-tRNAs. An *in silico* analysis of low-complexity TAPSLIVR-rich proteins functionally links this subset of the human proteome to cellular integrity, adhesion, and generation of, and interaction with the ECM, among others (Supplementary Table S5). We analysed endogenous expression of human transcripts encoding low-complexity TAPSLIVR-rich proteins in three different cellular systems. We detected translational defects in membrane-associated proteins such as LIPE, SPNS2, ORAI3, PIANP and SEMA6C (Figure 8A and Supplementary Table S4) (90), and in proteins containing MLDs (75–77) such as SDC3, Dystroglycan and MUC5AC (Figures 7D–F and Figure 9). Notably, not all low-complexity TAPSLIVR-rich proteins are transmembrane or secretory proteins (Supplementary Table S5), thus I34-tRNA depletion may affect translation of proteins both in the cytosol and the endoplasmic reticulum (91). The most striking translational phenotype caused by ADAT2 silencing was the reduction in the *de novo* synthesis of MUC5AC protein in NCI-H292 cells stimulated with AREG, despite a ∼1000-fold transcriptional activation of the *MUC5AC* gene (Figure 9D–H and Supplementary Figure S6B–E).

We also find that transcripts encoding low-complexity TAPSLIVR-rich proteins under high translational demand are more sensitive to I34-tRNA depletion. Our polysome profiling data detects translational impairment on transcripts encoding low-complexity TAPSLIVR-rich proteins that are highly translated in CTRL cells (Figure 8 and Supplementary Table S4). Likewise, we observe severe translational defects for MUC5AC in a cellular context where MUC5AC is required to be highly translated (i.e. upon AREG stimulation) (Figure 9D–H). On the other hand, translational impairment of SDC3 or Dystroglycan under standard growth conditions is milder (Figures 7D–F and 9A–C).

We determined that depletion of I34-tRNAs primarily affects translational efficiency, but not accuracy, of low-complexity TAPSLIVR-rich proteins, as seen by the time-dependent recovery of phenotypes (Figures 4D and 6A-B and Supplementary Figure S4A-B), time-course analysis of translation (Figure 7I), and mass spectrometry analyses (Supplementary Figure S5D). Furthermore, we showed that translation impairment is codon-dependent, as defects are not detected when TAPSLIVR codons are mutated to triplets not recognized by I34-tRNAs (Figure 7G–I). These results do not question a general role for I34-tRNAs in efficient translation (13,15), particularly because I34-tRNAs are required to decode all C-ended codons for TAPSLIVR (see Discussion above), a fact that explains why a full depletion of ADAT is lethal in all eukaryotic models (also this work, Figure 2A, B and see Materials and methods). Rather, our data support the hypothesis that a full complement of I34-tRNAs is essential for the efficient translation of low-complexity TAPSLIVR-rich coding sequences.

The fact that a partial downregulation of ADAT2 affects the translation of a specific subset of proteins without affecting overall protein fidelity or abundance could be used to develop new therapies designed to treat conditions caused by the accumulation of low-complexity TAPSLIVR-rich proteins, such as asthma or chronic obstructive pulmonary disease (92); or to control infection by viruses that may use MLDs for immunoevasion (93,94). Mutations in human ADAT have been associated to a complex syndrome that includes intellectual disability, microcephaly, and strabismus (95–100). The depletion of I34-tRNAs in our cellular models (Figure 2B and (17)) is similar to the reported levels of I34-tRNAs in patients carrying mutations in ADAT (97). Our results suggest that a defective synthesis of low-complexity TAPSLIVR-rich proteins might contribute to these phenotypes.

We characterized the phylogenetic distribution of homologs to human proteins with low-complexity TAPSLIVR-rich regions that depend on I34-tRNAs for their synthesis, and found that they are almost limited to eukaryotic species that abundantly utilize I34-tRNAs (Figure 10A, B and Supplementary Figure S7). It has previously been shown that tRNA genes encoding for I34-tRNA precursors (i.e. A34-tRNAs) are more abundant in eukaryotes than prokaryotes, a fact accompanied by a concomitant enrichment in eukaryotic codon usage bias towards codons cognate for I34-tRNAs (4,5,10,21,24,25). In addition, the abundance of A34-tRNA genes also correlates with the presence of TadA/ADAT required for A34-to-I34 editing (10,101–103). Here we show that, although I34-tRNAs exist in deeply-rooted eukaryotic groups (10,24,25), their abundance correlates with that of proteins with low-complexity TAPSLIVR-rich regions (Figure 10C, Supplementary Table S6 and Supplementary Figure S7). Strikingly, we find such proteins to be scarce in unicellular eukaryotes, with the sole exception of holozoan protists (the closest known relatives of metazoans (25)), where the abundance of low-complexity TAPSLIVR-rich proteins is comparable to that of multicellular species (Figure 10D-E and Supplementary Table S7)

Our data supports the hypothesis that I34-tRNAs contributed to expand eukaryotic proteome diversity, facilitating the synthesis of a specific set of low-complexity proteins involved in cellular interactions with the extracellular environment. There is evidence for adaptations of the translation machinery required for the synthesis of proteins of highly biased amino acid content. For example, the bacterial EF-P (eukaryotic eIF5A) is an elongation factor that allows the synthesis of poly-proline stretches (40). Likewise, in the salivary glands of certain arthropods, modulation of the tRNA pool is essential for the production of silk fibres that are alanine, glycine and serine rich (33). Other tRNA modifications have been reported important for decoding short stretches of consecutive codons (104,105). However, I34 is the first example of a translation machinery adaptation linked to the emergence of a new set of functionally-related proteins. We posit that the enrichment in I34-tRNAs provided organisms with the opportunity to translate low-complexity TAPSLIVR-rich proteins, which were then selected and expanded because of the functional advantages they provide in extracellular functions such as cellular adhesion. This is consistent with the proposal that unicellular ancestors to extant metazoans already possessed genetic features required for multicellularity (81–84). It is tempting to speculate that I34-tRNAs contributed to the burst of low-complexity TAPSLIVR-rich proteins in holozoan protists, which may have facilitated the advent of metazoan multicellularity.

## DATA AVAILABILITY

The datasets generated during this study are available at NCBI GEO (accession GSE150860), and at the ProteomeXchange Consortium via the PRIDE (dataset identifier PXD025024).

## Supplementary Material

gkab461_Supplemental_Files

## Notes

Present address: Àlbert Rafels-Ybern, National Centre for Genomic Analysis – Centre for Genomic Regulation (CNAG-CRG), Barcelona, Catalonia 08028. Spain.
