# Supplementary material for: Human tRNAs with inosine 34 are essential to efficiently translate eukarya-specific low-complexity proteins
Source: Nucleic Acids Res. 2021 Jun 14;49(12):7011–34. doi: 10.1093/nar/gkab461 (PMC8266599; doi:10.1093/nar/gkab461)
Supplement: gkab461_Supplemental_Files [file gkab461_supplemental_files.zip › 2021_Torres_etal_NAR_Suppl_data2.pdf]

**_Torres et al._ Human tRNAs with inosine 34 are essential to efficiently translate eukarya-specific low-complexity proteins. Supplementary Methods, Supplementary Figures and Legends for Supplementary Tables.**

## Supplementary Methods

### *Additional information on the generation of CRISPR-ADAT KD cell lines*

Sorting of GFP-positive cells was done by excitation of the sample using a 488 nm laser for forward scatter (FSC) and GFP (530nm); a 561 nm laser was used for the excitation of side scatter (SSC) signal. Single GFP expressing cells were selected according to the scatter and fluorescence parameters; doublets were excluded using the FSC-W signal.

To validate CRISPR cell lines (**Figure S1**).DNA was extracted using the DNeasy Blood and Tissue kit (69504, Qiagen) following the manufacturer’s protocol. Obtained DNA was quantified using a Nanodrop ND1000 and was used to PCR amplify ADAT genes. PCR amplifications were carried out using the Phusion High-Fidelity DNA Polymerase (F530S, Thermo Fisher) using primers depicted in **Supplementary Table S1**, and an annealing temperature of 60ºC for both set of primers. The obtained PCR products were gel-purified and sent for sequencing using as sequencing primers for the ADAT2 gene the same primers used for PCR amplification, and for ADAT3 another set of internal primers (see **Supplementary Table S1**).

### *Additional information on the cell line generation by lentiviral infection*

For hADAT2-pDONR221 plasmid generation, hADAT2 gene was amplified using oligonucleotides depicted in **Supplementary Table S1** (hADAT2 with attB sites) following a standard PCR reaction with pfu ultra polymerase (600384, Agilent). All PCR reactions described in this work were done in a MyCycler Thermal Cycler System (Bio-Rad). hADAT2 attB PCR product was purified and cloned into pDONR 221 vector (12536017, Invitrogen) following the manufacturer’s protocol (BP reaction).

For DOX-inducible shRNA expressing vectors the shRNA sequences were as follows:

shNonTarget sequence: 5’-TGCTGTTGACAGTGAGCGACCTCCACCCTCACTCTGCCATTAGTGAAGCCACAGATGTAATGGCAGAGTGAGGGTGGAGGGTGCCTACTGCCTCGGA-3’.

shADAT2 sequence: 5’-TGCTGTTGACAGTGAGCGCACACACTGTGTTGTATGTCACTAGTGAAGCCACAGATGTAGTGACATACAACACAGTGTGTTTGCCTACTGCCTCGGA-3’.

Lentivirus were produced in HEK293T cells growing in T75 flask format. Cells at approximately 80 % confluence were transfected with 75 µL of polyethylenimine (PEI) (23966-1, Polysciences), 1.5 µg envelope vector (VSVG), 1.5 µg Rev-expressing vector (RTR2), 4.5 µg packaging vector (PKG-PIR), 7.5 µg transfer vector and 1.5 mL 150 mM NaCl that was added to the cell culture media. 16 h later, cells were washed with PBS (14190094, Thermo Fisher), fresh Full Media was added and cells were left growing at 33 ºC/5 % $CO_2$. 48 hours post-transfection, viral supernatants were collected, cleared using a 0.45 µm filter, supplemented with 10 % FBS and 8 µg/mL polybrene (hexadimethrine bromide) (H9268, Sigma-Aldrich), and added to the cell line to be infected. Infected cells were left growing at 37 ºC/5 % $CO_2$. Fresh Full Media was added to the virus-producing HEK293T cells and were left growing at 33 ºC/5 % $CO_2$ for an additional day. The next day (72 hours post-transfection) viral supernatants were collected as before and were used to infect a second time the targeted cell line; at this point virus-producing cells were disposed. 24 hour after the second viral infection, cells were washed once with PBS and fresh Full Media was added. On the next day, 2 µg/mL puromycin (ant-pr-1, Invivogen) was added to culture medium for selection of transduced cells.

***Additional information on protein extraction***

Cells were harvested and washed once with 1 mL cold PBS. The cell pellet was re-suspended with an appropriate volume of RIPA buffer (50-250 µL). Cells were vortexed and incubated on ice for 30 minutes, followed by centrifugation at maximum speed at 4 ºC for 30 minutes. Supernatant (protein extract) was recovered and the remaining pellet was disposed.

***Antibodies used in this study***

Antibody used for western blotting and performed dilutions: PERK (C33E10) (3192, Cell Signaling Technology; RRID:AB_2095847) 1:500; IRE1 (phospho S724) (ab48187, Abcam; RRID:AB_873899) 1:1000; Phospho-eIF2α (Ser51) (9721, Cell Signaling Technology; RRID:AB_330951) 1:1000; eiF2α (9722, Cell Signaling Technology; RRID:AB_2230924) 1:1000; Actin (JLA20, Developmental Studies Hybridoma Bank; RRID:AB_528068) 1:1000; BiP (C50B12) (3177, Cell Signaling Technology; RRID:AB_2119845) 1:1000; ADAT2 (C-13) (sc-107385, Santa Cruz Biotechnology; RRID:AB_2273604) 1:1000; ADAT3 (N-term) (AP17369a, Abgent/Abcepta; RRID:AB_11136249) 1:200; Vinculin (SPM227) (ab18058, Abcam; RRID:AB_444215) 1:5000; GAPDH (G3PDH) (2275-PC-100, Trevigen; RRID:AB_2107456) 1:10000; Syndecan-3 (G-2) (sc-398194, Santa Cruz Biotechnology; RRID:AB_2732022) 1:500; Green Fluorescent Protein (06-896, Merck; RRID:AB_11214044) 1:1000; α-Dystroglycan (IIH6C4) (05-593, Merck; RRID:AB_309828) 1:250; β-Dystroglycan (MANDAG2(7D11)), Developmental Studies Hybridoma Bank; RRID:AB_2618140) 1:500; β-Tubulin (E7, Developmental Studies Hybridoma Bank; RRID:AB_528499) 1:1000; Phospho-EGF Receptor (Tyr1045) (2237, Cell Signaling Technology; RRID:AB_331710) 1:100; EGFR Antibody Cocktail (AHR5062, Thermo Fisher; RRID:AB_2536360) 1:250; Renilla Luciferase (PA5-32210, Thermo Fisher;

RRID:AB_2549683) 1:1000; Ubiquitin (Ub (P4D1)) (sc-8017, Santa Cruz Biotechnology; RRID:AB_628423) 1:1600. Secondary antibodies: Donkey Anti-Goat IgG H&L (HRP) (ab6885, Abcam; RRID:AB_955423) 1:10000; Sheep Anti-Mouse IgG (HRP) (NA931, VWR; RRID:AB_772210) 1:10000; Donkey Anti-Rabbit IgG (HRP) (NA934, VWR; RRID:AB_772206) 1:10000; Donkey Anti-Chicken IgY (HRP) (AP194P, Merck; RRID:AB_92682) 1:10000.

Antibodies used for protein purifications: Renilla luciferase antibody (PA5-32210, Thermo Fisher; RRID:AB_2549683); Green Fluorescent Protein antibody (DSHB-GFP-12A6, Developmental Studies Hybridoma Bank; RRID:AB_2617417).

Antibodies used for MUC5AC detection: MUC5AC (45M1) primary antibody (MA1-38223, Thermo Fisher; RRID:AB_2266697) diluted 1:200 and Anti-Mouse Alexa Fluor 555-conjugated secondary antibody (A-31570, Thermo Fisher; RRID:AB_2536180) for immunohistochemistry or Anti-Mouse Alexa Fluor 488-conjugated secondary antibody (A-21202, Thermo Fisher; RRID:AB_141607) for FACS analyses.

***Additional information on cell cycle analyses***

Cells growing on 10 cm Petri dish format at approximately 60 % confluence were incubated with 7 mL DMEM Full Media containing 2 mM Thymidine (T1895, Merck) for 13 hours at 37 ºC/5 % $CO_2$ (first thymidine cell cycle block). Media was then removed, cells were washed twice with 4 mL PBS, and were left growing in DMEM Full Media for 8 hours at 37 ºC/5 % $CO_2$. Media was then replaced by 7 mL DMEM Full Media containing 2 mM Thymidine and cells were left at 37 ºC/5 % $CO_2$ for 17hours (second thymidine cell cycle block). Cells were then washed twice with 4 mL PBS and were left growing on 6 mL of DMEM Full Media at 37 ºC/5 % $CO_2$ until harvesting time point.

Cells were harvested at time 0, 4, 8, and 12 hours after removal of the second thymidine block. Cells were detached using 5 mL PBS. Cell suspension was centrifuged for 3 minutes at 800 xg, the supernatant was removed and cells were re-suspended in 0.5 mL PBS. Then 4.5 mL ethanol 70 % was added to fix the cells and they were left at 4 ºC overnight.

The next day, the fixing solution was removed, cells were washed once with 5 mL PBS, and were re-suspended in 550 µL propidium iodide (PI) staining solution: 0.1 % Triton-X100, 2 mg/mL RNase A (Qiagen), 40 µg/mL PI (P4864), 1x PBS. Cells were incubated with staining solution for 3 hours at 37 ºC and were then analysed by FACS.

***Additional information on polysome profiling***

Sucrose gradients were generated in Open-top ultra clean tubes (344059, Beckman Coulter) with a Biocomp Gradient Station. 300 µL of cell lysate was loaded on top of the gradient and was centrifuged

using an SW41 rotor (Beckman Coulter) for 2 hours 30 minutes at 4 ºC at 35,000 RPM. After centrifugation the gradient was placed in the Gradient Station, scanned at 260 nm, and 20 fractions were collected per gradient. Gradient plots (**Figure 6A** and **Supplementary Figure S4A**) were normalized to the total 260 nm signal of the gradient to compensate for minor differences derived from unequal loading of cell lysates onto the gradient.

***Additional information on construction of ADAT eGFP reporters***

eGFP ADAT sequence:

GAATTCCTCGAGTCTAGAATGGTCAGCAAGGGCGAGGAGCTCTTCACCGGGGTCGTCCCCATCC
TCGTCGAGCTCGACGGCGACGTCAACGGCCACAAGTTCAGCGTCTCCGGCGAGGGCGAGGGC
GATGCCACCTACGGCAAGCTCACCCTCAAGTTCATCTGCACCACCGGCAAGCTCCCCGTCCCCT
GGCCCACCCTCGTCACCACCCTCACCTACGGCGTCCAGTGCTTCAGCCGCTACCCCGACCACAT
GAAGCAGCACGACTTCTTCAAGTCCGCCATGCCCGAAGGCTACGTCCAGGAGCGCACCATCTTC
TTCAAGGACGACGGCAACTACAAGACCCGCGCCGAGGTCAAGTTCGAGGGCGACACCCTCGTC
AACCGCATCGAGCTCAAGGGCATCGACTTCAAGGAGGACGGCAACATCCTCGGGCACAAGCTC
GAGTACAACTACAACAGCCACAACGTCTATATCATGGCCGACAAGCAGAAGAACGGCATCAAGG
TCAACTTCAAGATCCGCCACAACATCGAGGACGGCAGCGTCCAGCTCGCCGACCACTACCAGCA
GAACACCCCCATCGGCGACGGCCCCGTCCTCCTCCCCGACAACCACTACCTCAGCACCCAGTC
CGCCCTCAGCAAAGACCCCAACGAGAAGCGCGATCACATGGTCCTCCTCGAGTTCGTCACCGC
CGCCGGGATCACCCTCGGCATGGACGAGCTCTACAAGTAAGTTTAAACACCGGTGAATTC

eGFP nonADAT sequence:

GAATTCCTCGAGTCTAGAATGGTGAGCAAGGGCGAGGAGCTGTTCACGGGGGTGGTGCCGATC
CTGGTGGAGCTGGACGGCGACGTGAACGGCCACAAGTTCAGCGTGTCGGGCGAGGGCGAGGG
CGATGCGACGTACGGCAAGCTGACGCTGAAGTTCATCTGCACGACGGGCAAGCTGCCGGTGCC
GTGGCCGACGCTGGTGACGACGCTGACGTACGGCGTGCAGTGCTTCAGCCGGTACCCGGACCA
CATGAAGCAGCACGACTTCTTCAAGTCGGCGATGCCGGAAGGCTACGTGCAGGAGCGGACGAT
CTTCTTCAAGGACGACGGCAACTACAAGACGCGGGCGGAGGTGAAGTTCGAGGGCGACACGCT
GGTGAACCGGATCGAGCTGAAGGGCATCGACTTCAAGGAGGACGGCAACATCCTGGGGCACAA
GCTGGAGTACAACTACAACAGCCACAACGTGTATATCATGGCGGACAAGCAGAAGAACGGCATC
AAGGTGAACTTCAAGATCCGGCACAACATCGAGGACGGCAGCGTGCAGCTGGCGGACCACTAC
CAGCAGAACACGCCGATCGGCGACGGCCCGGTGCTGCTGCCGGACAACCACTACCTGAGCACG
CAGTCGGCGCTGAGCAAAGACCCGAACGAGAAGCGGGATCACATGGTGCTGCTGGAGTTCGTG
ACGGCGGCGGGGATCACGCTGGGCATGGACGAGCTGTACAAGTAAGTTTAAACACCGGTGAAT
TC

### *Additional information on evaluation of ADAT eGFP production*

For FACS analyses, excitation of the sample was done using a 488 nm air-cooled argon-ion laser at 15 mW power. The instrument was set up with the standard configuration: Forward scatter (FS), side scatter (SS), and green (525 nm) fluorescence for GFP. Fluorescence was collected in logarithmic scale. Optical alignment was checked using 10 nm fluorescent beads (Flow-Check fluorospheres, Catalog number 6605359; Beckman Coulter). Cell population was selected gating in a FS vs. SS dot plot, excluding aggregates and cell debris. To determine the percentage of GFP cells, a non-transfected control was used as negative reference.

Experiments depicted on **Supplementary Figure S5A-C** were performed on HEK293T DOX-inducible sh cell lines given that eGFP ADAT and eGFP nonADAT plasmids presented variable transfection efficiencies on HEK293T shCV and HEK293T shADAT2 cell lines. Cells were grown in DMEM Full Media with or without 1 μg/mL doxycycline (DOX) (D9891, Merck). At the time of lipofection, media from all cells was replaced and lipofection of eGFP ADAT and eGFP nonADAT plasmids was done without DOX. 4 hours after lipofection, cells were washed once with PBS and media was replaced by DMEM Full Media with or without DOX accordingly. 48 hours later cells were visualised in an Eclipse Ts2-FL microscope (Nikon).

### *Additional information on construction of ADAT luciferase reporters*

First, the desired portion of SDC3/SDC3(G-end) was PCR amplified using a forward (FWD) primer containing a NheI restriction site and a start codon; and a reverse (RVR) primer containing a BamHI restriction site and the NanoLuc linker. Next, the psiCHECK-2 RLuc gene was PCR amplified from the vector using a FWD primer containing a BamHI restriction site and a deletion of the ATG-start codon of the RLuc gene; and a RVR primer having a XhoI restriction site. All PCR amplifications were carried out using high-fidelity polymerases (e.g. pfu ultra polymerase). Both amplicons were gel-purified, digested with BamHI and ligated following standard procedures. The ligated product (insert) was further PCR amplified to obtain more material using primers SDC3 (or SDC3(G-end)) FWD and RLuc RVR, and the amplicon was gel-purified. The amplified insert and the parental psiCHECK-2 plasmid were digested with NheI and XhoI, and gel-purified (note that this digestion removes the RLuc gene from the psiCHECK-2 plasmid). Digested vector and insert where then ligated using standard procedures to incorporate the SDC3-RLuc or SDC3(G-end)-RLuc insert into the vector.

SDC3 wild type low-complexity TAPSLIVR-rich region nucleotide sequence:

GAAGAGCTCCCCTCTGAGCGCCCCACCCTGGAGCCAGCCACCAGCCCCCTGGTGGTGACAGAA
GTCCCGGAAGAGCCCAGCCAGAGAGCCACCACCGTCTCCACTACCATGGCTACCACTGCTGCC
ACAAGCACAGGGGACCCGACTGTGGCCACAGTGCCTGCCACAGTGGCCACCGCCACCCCCAGC
ACCCCTGCAGCACCCCCTTTTACGGCCACCACTGCTGTTATAAGGACCACTGGCGTACGGAGGC
TTCTGCCTCTCCCACTGACCACAGTGGCTACGGCACGGGCCACTACCCCCGAGGCGCCCTCCC

CGCCCACCACGGCGGCTGTCTTGGACACCGAGGCCCCAACACCCAGGCTGGTCAGCACAGCTA
CCTCCCGGCCAAGAGCCCTTCCCAGGCCGGCCACCACCCAGGAGCCTGACATCCCTGAGAGGA
GCACCCTGCCCCTGGGGACCACTGCCCCTGGACCCACAGAGGTGGCTCAGACCCCAACTCCAG
AGACCTTCCTGACCACA

SDC3(G-end) low-complexity TAPSLIVR-rich region nucleotide sequence:

GAAGAGCTGCCGTCGGAGCGGCCGACGCTGGAGCCGGCGACGAGCCCGCTGGTGGTGACGGA
AGTGCCGGAAGAGCCGAGCCAGAGAGCGACGACGGTGTCGACGACGATGGCGACGACGGCGG
CGACGAGCACGGGGGACCCGACGGTGGCGACGGTGCCGGCGACGGTGGCGACGGCGACGCC
GAGCACGCCGGCGGCGCCGCCGTTTACGGCGACGACGGCGGTGATAAGGACGACGGGCGTGC
GGAGGCTGCTGCCGCTGCCGCTGACGACGGTGGCGACGGCGCGGGCGACGACGCCGGAGGC
GCCGTCGCCGCCGACGACGGCGGCGGTGTTGGACACGGAGGCGCCGACGCCGAGGCTGGTG
AGCACGGCGACGTCGCGGCCGAGAGCGCTGCCGAGGCCGGCGACGACGCAGGAGCCGGACA
TACCGGAGAGGAGCACGCTGCCGCTGGGGACGACGGCGCCGGGACCGACGGAGGTGGCGCA
GACGCCGACGCCGGAGACGTTCCTGACGACG

***Additional information on purification of reporter proteins***

HEK293T cells growing in 150 mm Petri dish format at around 90 % confluence were transfected with 10 µg SDC3-RLuc or SDC3(G-end)-RLuc constructs using L2K following the manufacturer's protocol (500 µL plasmid/lipid reaction in 21 mL DMEM Full Media). 48 hours after lipofection, cells were washed once with 5 mL cold PBS and were then collected into a pre-chilled 10 mL Falcon tube, by cell-scrapping using 3 mL cold PBS. Cells were then centrifuged at 500 xg for 3 minutes at 4 ºC, supernatant was removed and cell pellets were washed once with 3 mL cold PBS. Cells were centrifuged again, supernatant was removed and cell pellets were re-suspended in 500 µL Lysis buffer (20 mM Tris pH 8, 2 mM EDTA, 1 % NP-40, 150 mM NaCl, 0.1 mM NaVaO, 1X PIC), and incubated in a rotating wheel for 30 minutes at 4 ºC. Cells were then centrifuged at maximum speed at 4 ºC for 30 minutes and the supernatant was recovered and transferred to a new pre-chilled tube ("cell lysate"). Cell lysate was incubated with 20 µL magnetic Dynabeads Protein A (10002D, Thermo Fisher) in a rotating wheel for 2 hours at 4 ºC. Beads were separated using a magnet and the supernatant was recovered and transferred to a new pre-chilled tube ("pre-cleared cell lysate"). 50 µL dynabeads A were incubated for 10 minutes in a rotating wheel at RTº with 5 µg Renilla luciferase antibody (PA5-32210, Thermo Fisher) in 200 µL Wash Buffer (100 mM Tris pH 7.5, 1 mM EDTA, 1 mM EGTA, 1 % NP-40, 350 mM NaCl, 0.2 mM NaVaO, 1x PIC) ("Ig-Dynabeads"). Supernatant was removed and Ig-Dynabeads were washed twice with 200 µL Wash Buffer. Ig-Dynabeads were then washed twice with 200 µL Conjugation Buffer and were then cross-linked by re-suspending them in freshly prepared 250 µL 5 mM $BS^3$ (21580, Thermo Fisher) in Conjugation Buffer, and incubating them for 30 minutes at RTº in a rotating wheel. Then, 12.5 µL of 1 M Tris pH 7.5 was added to the reaction, which was further incubated for 15 minutes at RTº in a rotating wheel to quench it. Cross-linked Ig-Dynabeads were then washed 3 times with 200

µL Wash Buffer and were left overnight incubating with pre-cleared cell lysate at 4 ºC in a rotating wheel. The next day, beads were recovered and washed twice with 500 µL Wash Buffer. Elution was performed by incubating beads with 40 µL 0.25 % trifluoroacetic acid (TFA) for 5 minutes at RTº. The solution was then neutralized with Tris pH 8. Eluted samples were mixed with Protein Loading Buffer (100 mM Tris pH 6.8, 4 % SDS, 0.1 % Bromophenol Blue, 20 % Glycerol, 100 mM DTT) and resolved in a 10 % PAGE that was further stained with BlueSafe. Protein bands corresponding to the SDC3-RLuc and SDC(G-end)-RLuc were gel purified and submitted to mass spectrometry analyses.

Purification of eGFP reporter proteins was performed essentially as described above but using Protein G sepharose beads (17-0618-01, VWR). Cells in lysis buffer were kept in ice and were disrupted by passing them through a 27G syringe five times. Cells were then centrifuged for 20 minutes at maximum speed at 4 ºC and the supernatant was recovered ("cell lysate"). 100 µL Protein G sepharose beads were equilibrated with 2 volumes of lysis buffer, centrifuged for 2 minutes at 700 xg at 4 ºC and re-suspended in lysis buffer for a final 50 % vol/vol. Cell lysate was incubated with 30 µL of equilibrated Protein G sepharose beads (eqProtG) in a rotating wheel for 2 hours at 4 ºC, and was then centrifuged for 20 minutes at 700 xg at 4 ºC. Supernatant was recovered ("pre-cleared cell lysate"). 50 µL eqProtG were incubated for 1 hour in a rotating wheel at RTº with 5 µg Green Fluorescent Protein antibody (DSHB-GFP-12A6, Developmental Studies Hybridoma Bank) in 200 µL lysis buffer ("Ig-eqProtG"). Ig-eqProtG were then incubated with pre-cleared cell lysate overnight at 4 ºC in a rotating wheel. The next day, samples were centrifuged for 10 minutes at 400 xg at 4 ºC, and beads were washed twice with 1 mL ice-cold lysis buffer. Elution was carried out by re-suspending the beads in 40 µL SDS sample buffer 1x (50 mM Tris pH 6.8, 2 % SDS, 0.1 % Bromophenol blue, 10 % glycerol) and incubating them for 15 minutes at 50 ºC. Samples were then centrifuged for 1 minute at 1000 xg at RTº and 10 % DTT was added to the eluted samples. Proteins were resolved in a 10 % PAGE, gel was stained with BlueSafe and bands corresponding to eGFP were gel purified and submitted to mass spectrometry analyses.

**Supplementary Figures**

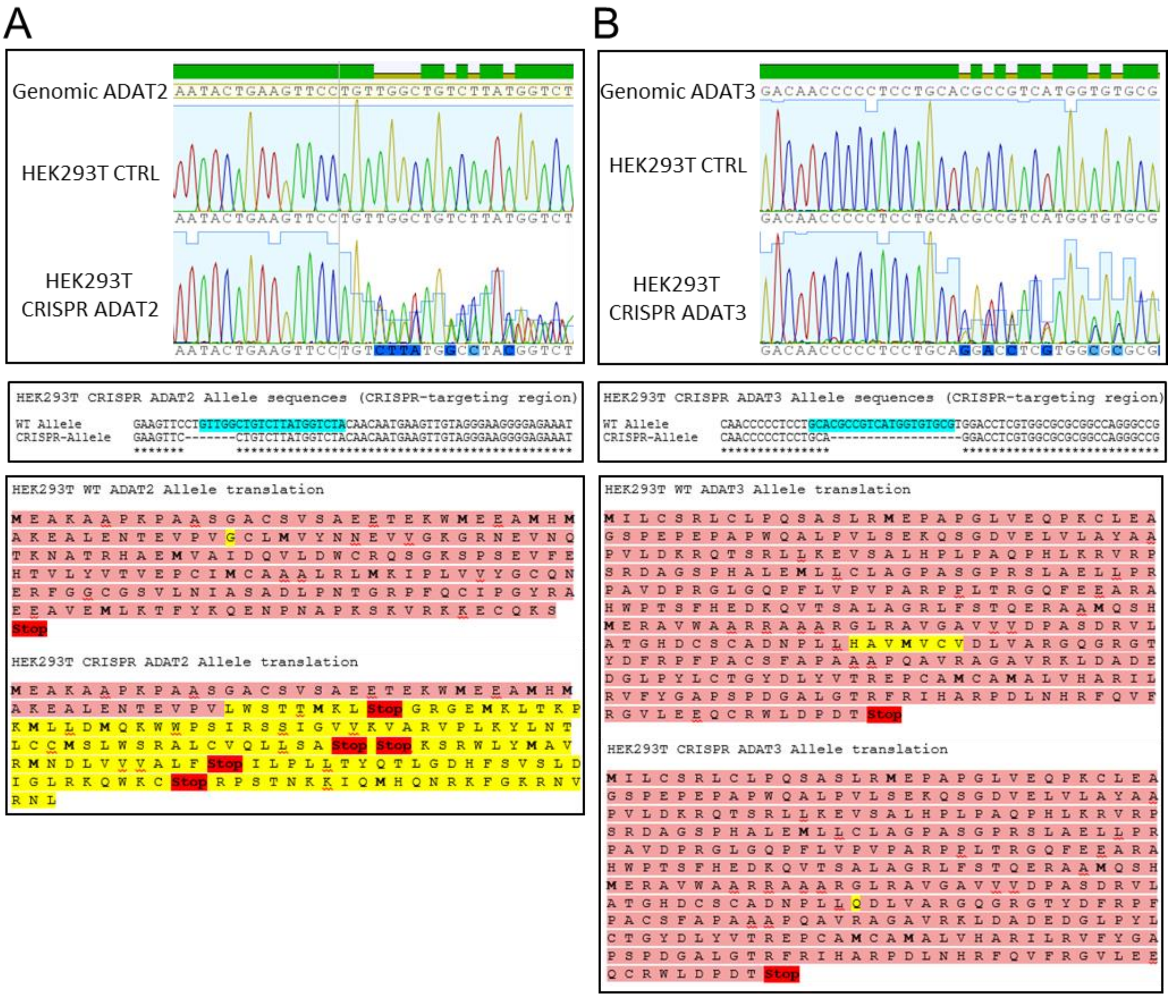


**Supplementary Figure S1**. (A) and (B) Upper panels: Sanger-sequencing spectra for amplified genes in HEK293T CTRL, and HEK293T ADAT2 KD (A) or HEK293T ADAT3 KD (B) cells. Centre panels: Sequence alignments of wild type and CRISPR/Cas9-edited alleles. Shaded sequence correspond to the section targeted by the guide strands. Lower panels: predicted translation of wild type and CRISPR/Cas9-edited alleles. Affected regions are highlighted in yellow. The deletion in the ADAT2 gene causes a frame-shift leading to a premature STOP codon. The deletion in the ADAT3 gene removes part of the deaminase domain.

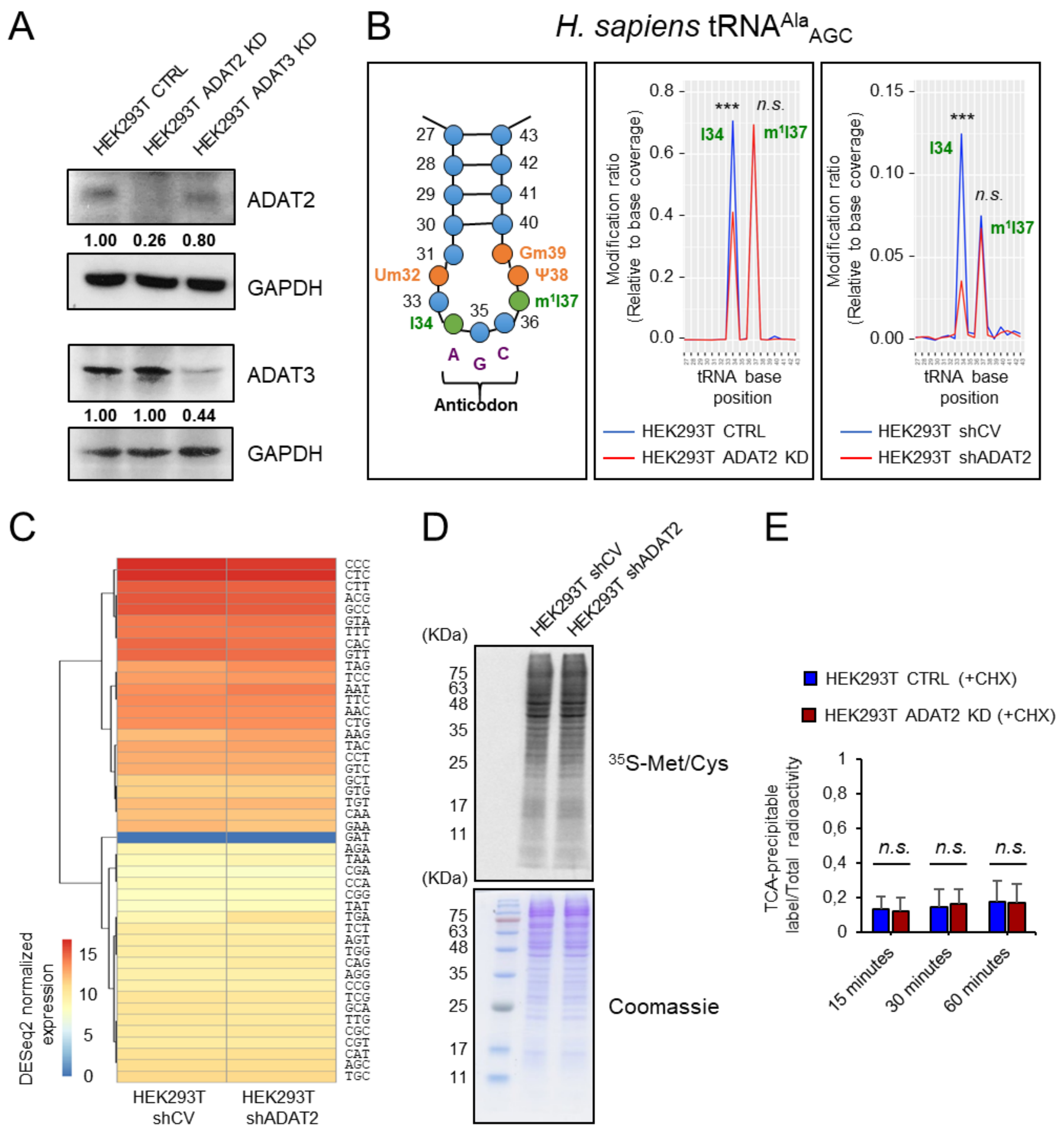


**Supplementary Figure S2**. (A) ADAT2 and ADAT3 protein levels evaluated by western blotting in the indicated cell lines. GAPDH is used as gel loading control. Quantification of gel bands relative to GAPDH and normalized to HEK293T CTRL cells are shown. (B) Left panel: schematic representation of the anticodon stem-loop structure of *Homo sapiens* $tRNA^{Ala}_{AGC}$ depicting known modified residues. Modifications detected (green) and not detected (orange) by tRNA-Seq are shown. Numbers indicate the tRNA base position. Anticodon position and sequence (AGC) is shown for reference (purple). The first anticodon position (A34) is deaminated to I34 (see also **Figure 1A**). Um (2′-O-methyluridine); I (Inosine); $m^1I$ (1-methylinosine); Ψ (Pseudouridine); Gm (2′-O-methylguanosine). Centre and Right panels: modification ratio (proportion of sequencing mismatches relative to base sequencing coverage) observed in sequencing reads mapping to human $tRNA^{Ala}_{AGC}$ genes as seen by tRNA-Seq in the indicated cell lines. The expected tRNA modification at the indicated base position (I34 and $m^1I37$) is shown in green for reference (see also left panel). Centre panel: modification ratio in HEK293T CTRL

(blue) and HEK293T ADAT2 KD (red) cells. Right panel: modification ratio in HEK293T shCV (blue) and HEK293T shADAT2 (red) cells (see also (17)). *n.s.*: not statistically significant. ***: adj. p-val < 0.001 (Benjamini-Hochberg, Fisher Exact test). (C) Heatmap visualization of tRNA gene expression at isodecoder level (tRNAs with the same anticodon) in HEK293T shCV and shADAT2 cells as evaluated by tRNA-Seq. Colouring scale represents log2 DESeq2 normalized expression values based on two biological replicates calculated as in (51). No statistically significant differences were found (Benjamini-Hochberg). (D) Pulse-chase analyses on HEK293T shCV and shADAT2 cells. Coomassie staining of the same gel is used as loading control. (E) Quantitative evaluation of $^{35}$S-Met/Cys incorporation into proteins in the presence of cycloheximide (CHX) upon monitoring the ratio between the total radioactivity (cpm) of the cell lysate and the amount of radioactivity (cpm) in TCA-precipitable fractions. Shown are the mean and standard deviations from biological triplicates. *n.s.*: not statistically significant (t-test). See also **Figure 3C**.

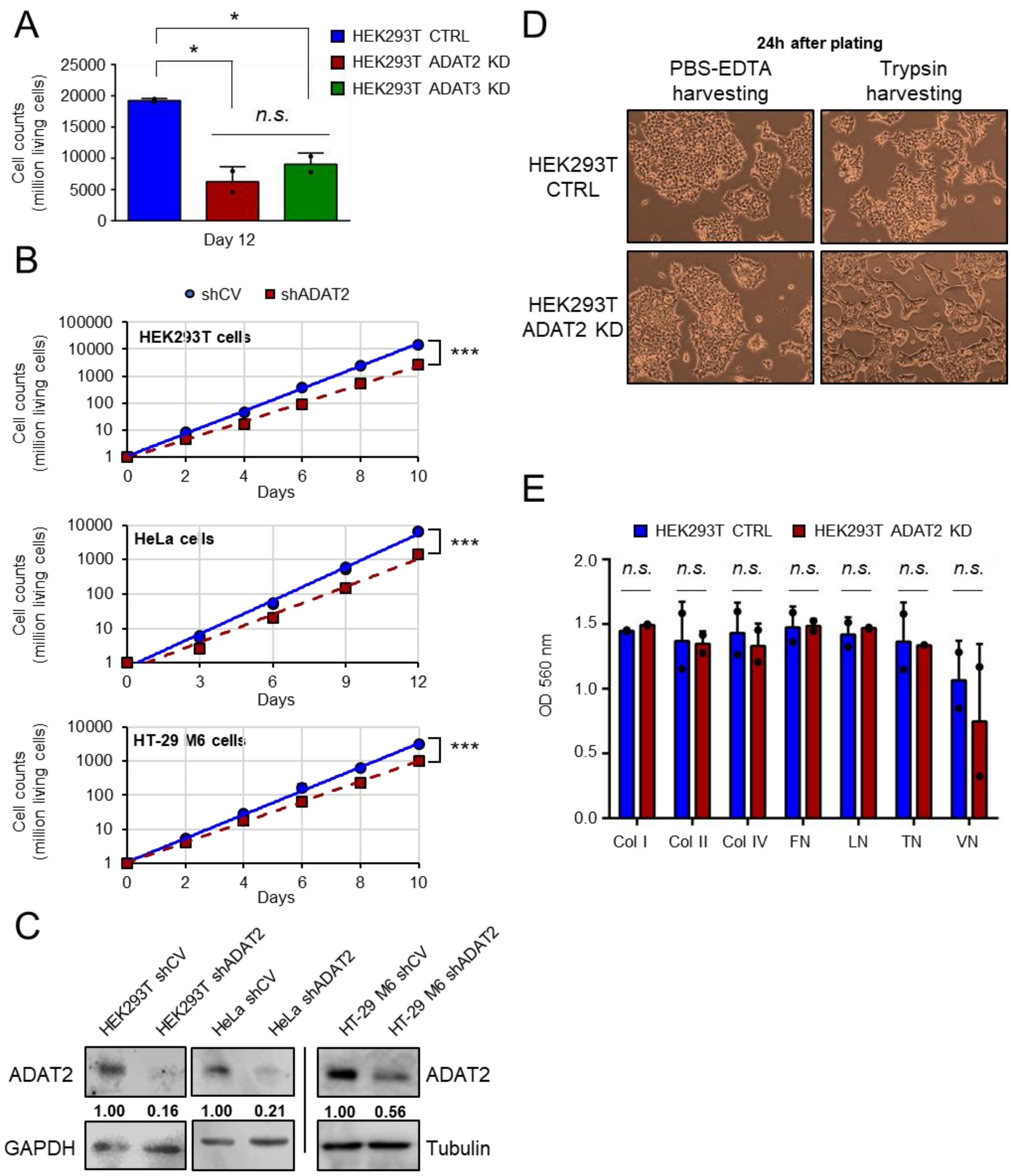


**Supplementary Figure S3**. (A) Comparative analysis of cell growth at Day 12, represented as total cell counts, for the indicated cell lines. Shown are biological duplicates, their mean and SD. *n.s.*: not statistically significant. *: p-val < 0.05 (t-test). (B) Growth curves of HEK293T, HeLa and HT-29 M6 shCV (blue) and shADAT2 (red) cells represented as total counts of living cells over time. Y-axis is set in logarithmic scale and an exponential trendline was fit to the data points. Data points correspond to the mean and SD of three biological replicates. ***: p-val < 0.001 (t-test). (C) ADAT2 protein levels evaluated by western blotting in the indicated cell lines. GAPDH or Tubulin is used as gel loading control. Quantification of ADAT2 bands relative to GAPDH or Tubulin and normalized to shCV cells is shown (D) Representative light microscopy images of HEK293T CTRL and ADAT2 KD cells harvested

with PBS-EDTA (left panels) or Trypsin (right panels) at 24 hours after plating them in clean culture plates. (E) Evaluation of cell adhesion capacity to components of the extracellular matrix when cells are collected for this analysis with trypsin instead of PBS-EDTA (cell adhesion profiles are expected to be similar for both cell lines because newly synthesised adhesion proteins are removed using trypsin). Col I, II and IV: collagen I, II and IV respectively. FN: fibronectin. LN: laminin. TN: tenascin. VN: vitronectin. Data shows the mean and SD of the obtained absorbance at 560 nm (OD 560 nm) for HEK293T CTRL (blue) and ADAT2 KD (red) cells. Experiments were done in biological duplicates. *n.s.*: not statistically significant (t-test).

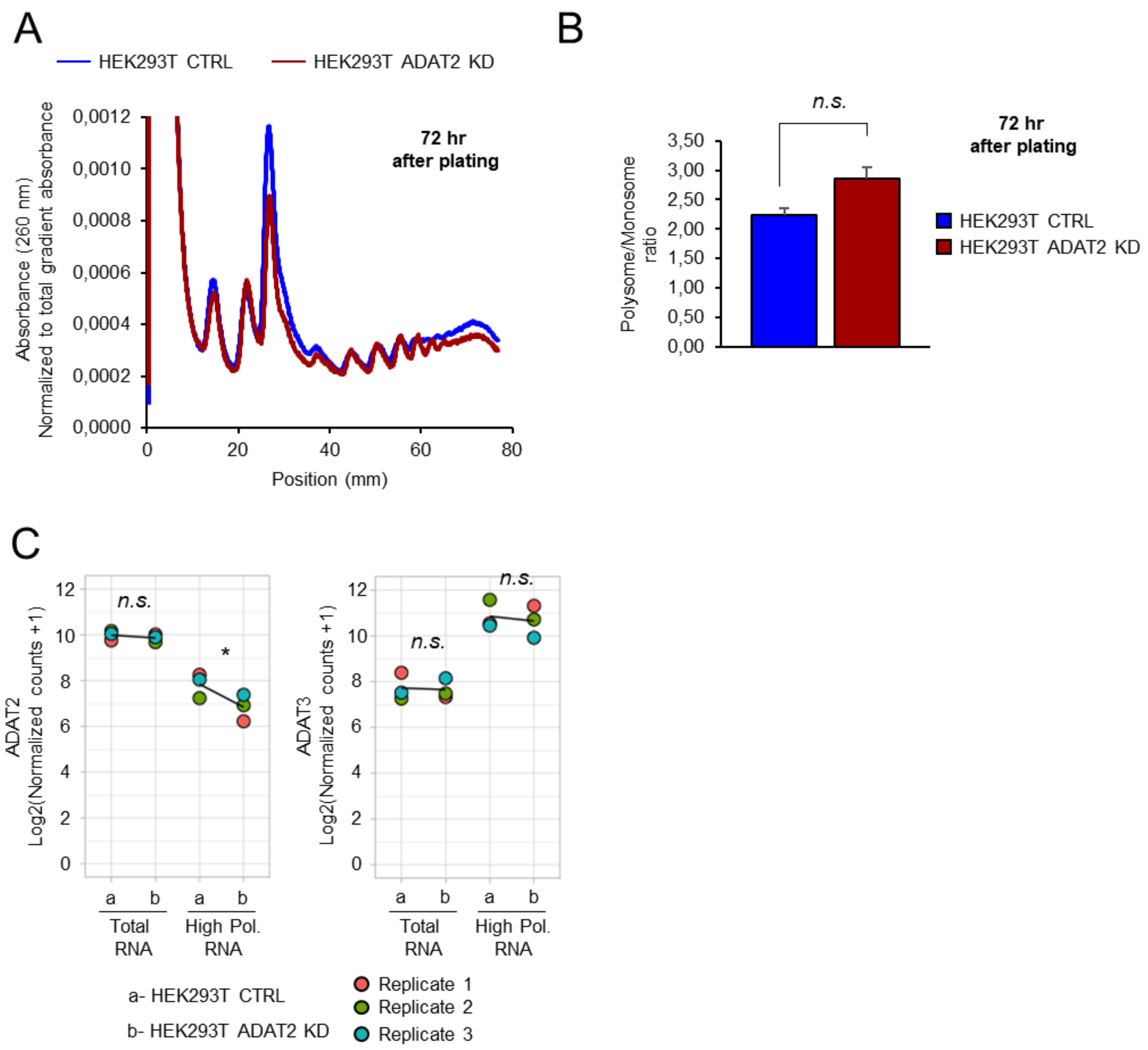


**Supplementary Figure S4**. (A) Representative polysome profile of trypsin-treated HEK293T CTRL (blue) and ADAT2 KD (red) cells at 72 hours after plating. Experiments were done in biological triplicates. (B) Polysome to Monosome ratio (P/M) obtained from experiments as in (A). Shown are the mean and standard deviations of biological triplicates. *n.s.*: not statistically significant (t-test). (C) Quantification of ADAT2 (left panel) and ADAT3 (right panel) transcript abundance (sequencing reads: log2(normalized counts + 1)) in Total RNA and the HP fractions on the indicated cell lines. Dots represent the obtained quantification for each biological triplicate. The line connects the means of the compared groups. *n.s.*: not statistically significant. *: adj. p-val < 0.1 (DESeq2, Benjamini-Hochberg).

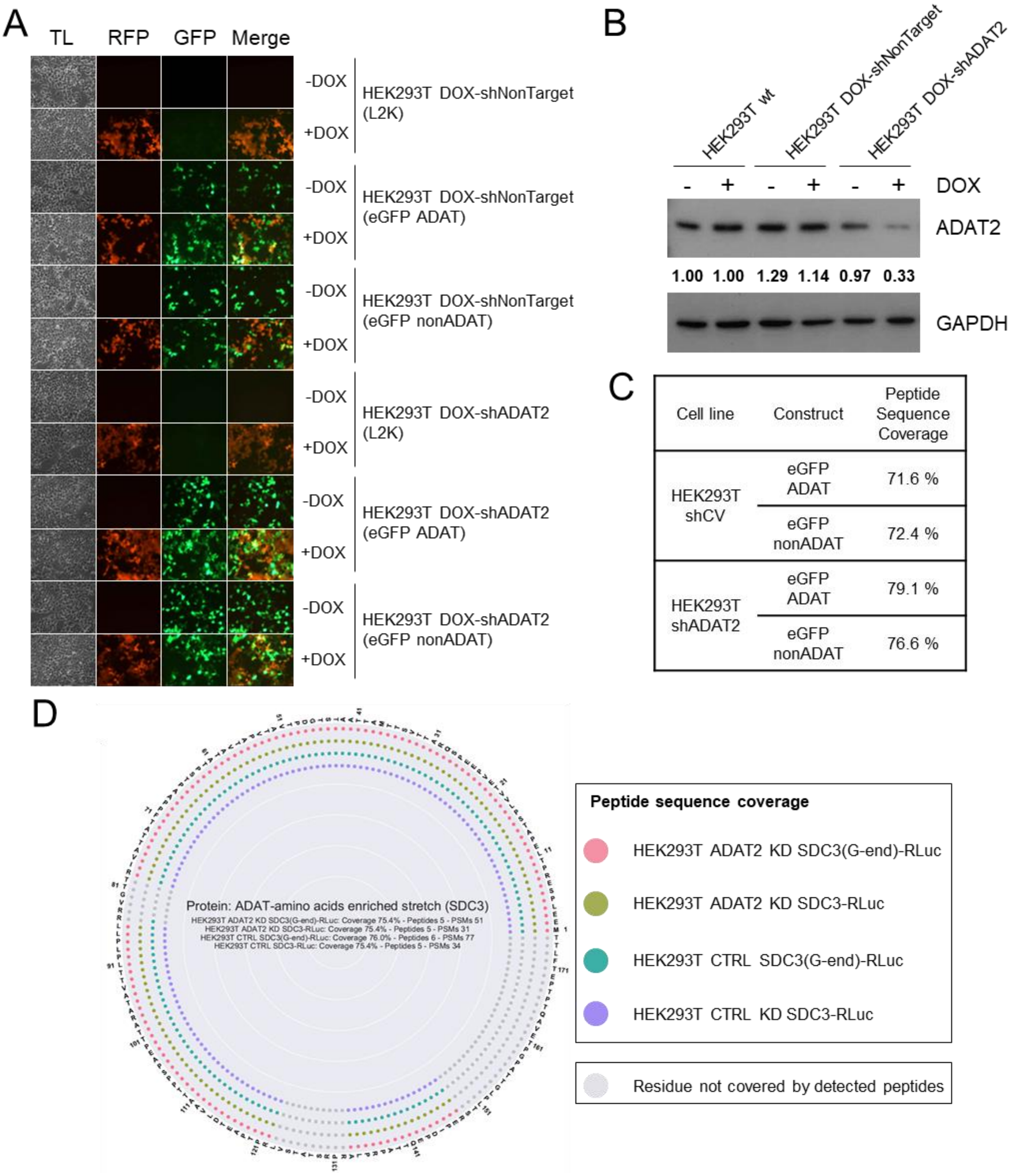


| Cell line | Construct | Peptide Sequence Coverage |
|---|---|---|
| HEK293T shCV | eGFP ADAT | 71.6 % |
| | eGFP nonADAT | 72.4 % |
| HEK293T shADAT2 | eGFP ADAT | 79.1 % |
| | eGFP nonADAT | 76.6 % |

**Supplementary Figure S5**. (A) Transmittance light (TL) and epifluorescence (Red Fluorescent Protein, RFP, and Green Fluorescent Protein, GFP) microscopy images of doxycycline (DOX)-inducible ADAT KD cell lines transfected with eGFP ADAT or eGFP-nonADAT constructs. Addition of DOX (+) activates the generation of the inducible shRNA coupled to production of RFP. (B) ADAT2 protein levels evaluated by western blotting in the indicated cell lines in the presence (+) or absence (-) of doxycycline (DOX). GAPDH is used as gel loading control. Quantification of ADAT2 bands relative to GAPDH and normalized to WT cells with DOX (for (+) DOX treatments) or without DOX (for (-) DOX treatments) is shown. (C) Percentage of eGFP peptide sequence coverage as detected by mass spectrometry of purified eGFPs obtained upon expression of eGFP ADAT or eGFP nonADAT constructs in the indicated cell lines. (D) Evaluation of mistranslation of the low-complexity TAPSLIVR-rich region of SDC3 by *de*

*novo* protein sequencing of purified SDC3-RLuc or SDC3(G-end)-RLuc expressed in HEK293T CTRL or ADAT2 KD cells. The wheel represents the full sequence of the TAPSLIVR-rich region of SDC3. Amino acids covered by the obtained peptides by mass spectrometry in each sample are color-coded. Grey circles are undetected residues. Percentages of sequence coverage for each sample are shown.

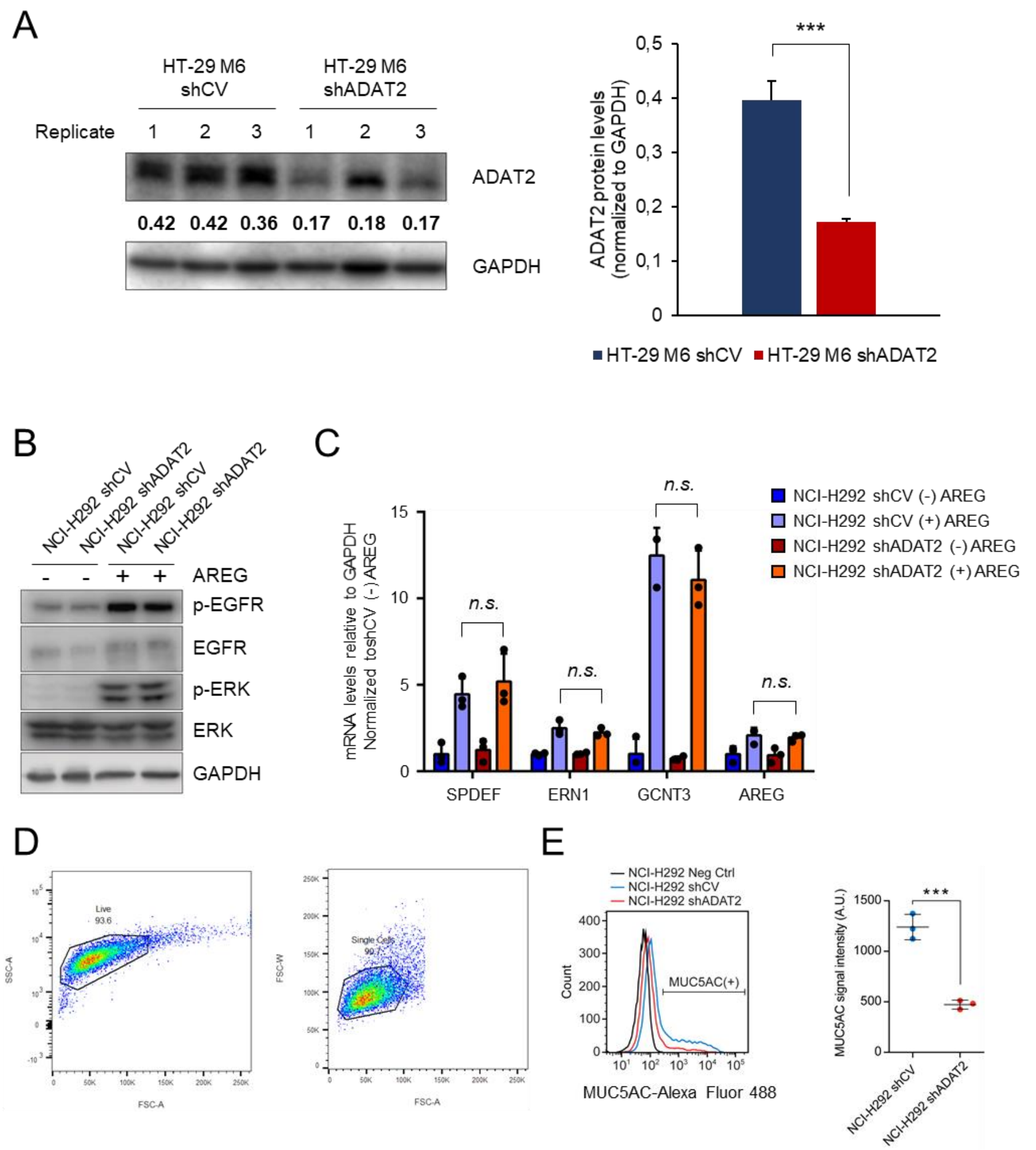


Supplementary Figure S6. (A) ADAT2 protein levels in extracts depicted in **Figure 9B**. Quantification of ADAT2 bands relative to GAPDH is shown. Bar plot represents the mean and SD obtained from quantifying the shown blots (biological triplicates). ***: p-val < 0.001 (t-test) (B) Evaluation by western blotting of the expression of markers of AREG stimulation in the indicated cell lines treated (+) or not (-) with AREG. GAPDH is used as gel loading control. (C) Real time qPCR of markers of AREG stimulation relative to GAPDH in the indicated cell lines treated (+) or not (-) with AREG. Data is normalized to untreated NCI-H292 shCV cells for each target gene. Shown are biological triplicates, their mean and SD. Statistical significance is shown only for the comparisons between NCI-H292 shCV and shADAT2 in the presence of AREG (both cell lines show similar transcriptional activation of the evaluated targets upon AREG treatments). *n.s.*: not statistically significant (t-test). (D) Flow cytometry analysis of the experiments depicted in **Figure 9G** showing representative pseudocolor plots exemplifying the gating strategy used, and including both MUC5AC(+) and MUC5AC(-) cells. (E) Left

panel: NCI-H292 negative control cells (black line; cells incubated only with the secondary antibody) was used to set up the MUC5AC(+) gating (shown in figure). NCI-H292 shCV cells shown in blue and NCI-H292 shADAT2 cells shown in red. Right panel: quantification of MUC5AC signal intensity as shown in **Figure 9G** but including the signal obtained for the whole live/single cell suspension (i.e. without MUC5AC(+) gating).

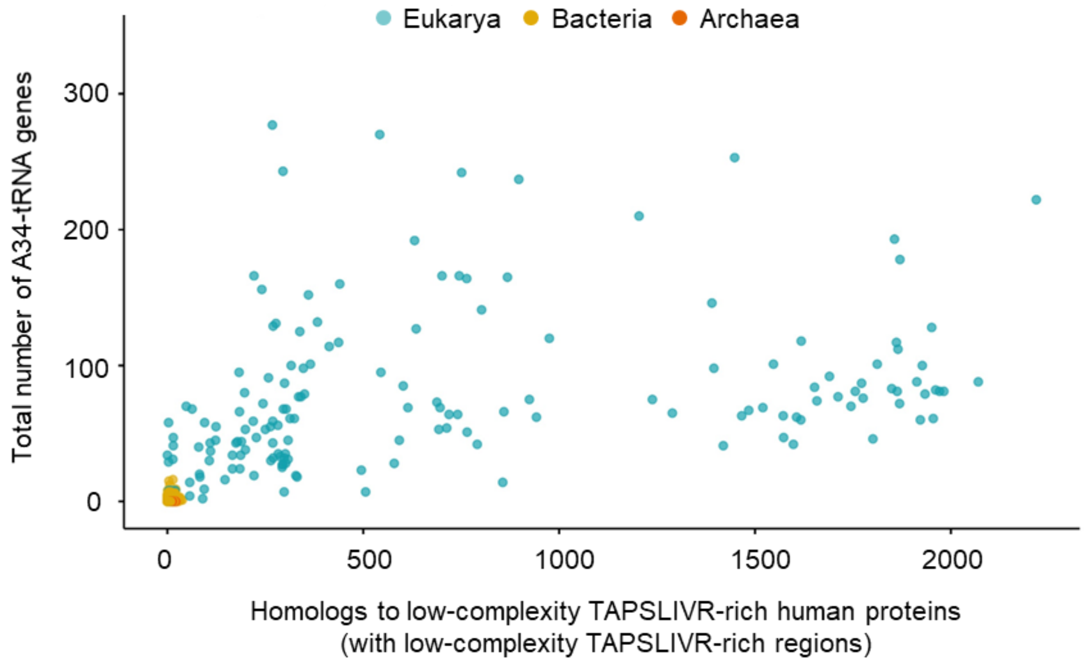


Supplementary Figure S7. Distribution of species from Eukarya (turquoise), Bacteria (yellow) and Archaea (dark orange) based on the total number of A34-tRNA genes and the number of homologs to low-complexity TAPSLIVR-rich human proteins present in their genomes. Note that the data on eukaryotic species is the same one depicted on **Figure 10C,** and is used here for reference.

**Legends for Supplementary Tables**

Supplementary Table S1. Oligonucleotides used in this study.

Supplementary Table S2. Differential tRNA gene expression analyses in HEK293T CTRL, HEK293T ADAT2 KD, HEK293T shCV, and HEK293T shADAT2 cell lines.

Supplementary Table S3. Proteomic analyses on HEK293T CTRL and HEK293T ADAT2 KD (iTRAQ). Table with fold changes, p-values, mean estimates and confidence intervals.

Supplementary Table S4. Differential transcript expression in Total RNA and RNA from High Polysome fractions between HEK293T CTRL and HEK293T ADAT2 KD cells; GSEA (GO Biological Process); Interaction analysis and Fisher Exact Test for proportion of transcripts encoding low-complexity TAPSLIVR-rich regions with impaired translation upon ADAT2 KD.

Supplementary Table S5. Identified human proteins containing low-complexity TAPSLIVR-rich regions, and GO analyses obtained by DAVID (59).

Supplementary Table S6. Abundance of A34-tRNA genes and number of low-complexity TAPSLIVR-rich human proteins with homologs in eukaryotic and prokaryotic species. Highlighted eukaryotic species contain an unusually large (> 400) number of A34-tRNA genes and were removed from the analyses (See also *Materials and methods*).

Supplementary Table S7. Abundance of homologous sequences to human low-complexity TAPSLIVR-rich proteins in unicellular and multicellular organisms.
